# Fully automated measurement of plasma Aβ42/40 and p‐tau181: Analytical robustness and concordance with cerebrospinal fluid profile along the Alzheimer's disease continuum in two independent cohorts

**DOI:** 10.1002/alz.13687

**Published:** 2024-02-07

**Authors:** Giovanni Bellomo, Sherif Bayoumy, Alfredo Megaro, Andrea Toja, Giovanna Nardi, Lorenzo Gaetani, Elena R. Blujdea, Federico Paolini Paoletti, Anna Lidia Wojdaƚa, Davide Chiasserini, Wiesje M. van der Flier, Inge M. W. Verberk, Charlotte Teunissen, Lucilla Parnetti

**Affiliations:** ^1^ Center for Memory Disturbances Lab of Clinical Neurochemistry Section of Neurology Department of Medicine and Surgery University of Perugia Perugia Italy; ^2^ Neurochemistry Laboratory Department of Laboratory Medicine Amsterdam Neuroscience, Amsterdam UMC Amsterdam The Netherlands; ^3^ Section of Biochemistry Department of Medicine and Surgery University of Perugia Perugia Italy; ^4^ Alzheimer Center Department of Neurology Vrije Universiteit Amsterdam, Amsterdam UMC Amsterdam The Netherlands; ^5^ Department of Epidemiology and Data Science Vrije Universiteit Amsterdam Amsterdam UMC Amsterdam The Netherlands

**Keywords:** Aβ, Alzheimer's disease, automated platforms, plasma biomarkers, p‐tau

## Abstract

**INTRODUCTION:**

For routine clinical implementation of Alzheimer's disease (AD) plasma biomarkers, fully automated random‐access platforms are crucial to ensure reproducible measurements. We aimed to perform an analytical validation and to establish cutoffs for AD plasma biomarkers measured with Lumipulse.

**METHODS:**

Two cohorts were included. UNIPG: *n* = 450 paired cerebrospinal fluid (CSF)/plasma samples from subjects along the AD‐continuum, subjects affected by other neurodegenerative diseases, and controls with known CSF profile; AMS: *n* = 40 plasma samples from AD and *n* = 40 controls. Plasma amyloid β (Aβ)42, Aβ40, and p‐tau181 were measured with Lumipulse. We evaluated analytical and diagnostic performance.

**RESULTS:**

Lumipulse assays showed high analytical performance. Plasma p‐tau181 levels accurately reflected CSF A+/T+ profile in AD‐dementia and mild cognitive impairment (MCI)‐AD, but not in asymptomatic‐AD. Plasma and CSF Aβ42/40 values were concordant across clinical AD stages. Cutoffs and probability‐based models performed satisfactorily in both cohorts.

**DISCUSSION:**

The identified cutoffs and probability‐based models represent a significant step toward plasma AD molecular diagnosis.

## 1 BACKGROUND

Cerebrospinal fluid (CSF) biomarkers play a crucial role in early diagnosis of Alzheimer's disease (AD).^1^, ^2^ According to the National Institute on Aging and Alzheimer's Association (NIA‐AA) criteria,^3^ AD could be diagnosed independently from the clinical stage, in the presence of abnormally low CSF amyloid β (Aβ)42/40 ratio (A+), and increased phosphorylated tau (T+).

Over the past decade, significant effort has been made to enable the measurement of AD biomarkers in blood, with clear advantages in terms of reduced invasiveness, cost, and accessibility. The ultrasensitive measurement of plasma Aβ42/40 and p‐tau181 demonstrated a satisfactory agreement with CSF AD biomarkers and a strong association with clinical symptoms.^4^, ^5^ However, high inter‐center and inter‐assay variability have been observed,^6^, ^7^, ^8^ hampering the calculation of reliable cutoff values for the clinical use of plasma AD biomarkers.

Considering an urgent need to implement plasma AD biomarkers into clinical practice, the adoption of automated systems that are cost‐effective, easy to use, feasible for use on single samples, and already available for the measurement of CSF biomarkers could be an optimal solution.^2^, ^9^ To this end, chemiluminescent enzyme immunoassays (CLEIA) for plasma Aβ42/40 and p‐tau181 measurements have been developed on a fully automated platform (Lumipulse). In this study, we aimed to assess the analytical performance of Lumipulse G600II assays for plasma Aβ42/40 and p‐tau181, and the concordance with CSF values by also considering potential confounders such as kidney dysfunction (KD) and blood‐brain barrier (BBB) permeability. These measurements were performed in a wide range of clinical conditions, such as controls, the full spectrum of clinical stages of AD, Parkinson's disease (PD), dementia with Lewy bodies (DLB), and frontotemporal dementia (FTD). Finally, using independent training and validation cohorts, we determined cutoff values and probability ranges for plasma Aβ42/40 and p‐tau181 for identification of CSF A+ and A+/T+ profiles.

## 2 METHODS

### 2.1 Study participants

#### 2.1.1 UNIPG cohort

The cohort was selected from a consecutive series of individuals (*n* = 450) referred to the Centre of Memory Disturbances of the University Hospital of Perugia, Perugia, Italy, between 2015 and 2021. All of them underwent a comprehensive assessment including medical history, physical, and neurological examination, laboratory tests, and a thorough neuropsychological evaluation. Brain imaging, either computed tomography or magnetic resonance imaging, was performed in all cases.^18^Fluoro‐2‐deoxyglucose positron emission tomography (^18^F‐FDG‐PET) and dopamine transporter scan (DaT‐Scan) were also performed in selected cases, based on clinical judgement. All patients underwent CSF analysis for the measurement of CSF Aβ42/40, p‐tau181, and total tau (t‐tau) levels, using automated Lumipulse technology. Therefore, CSF A/T profiles of all subjects were available, according to cutoffs developed at *UNIPG* for CSF Aβ42/40 and p‐tau181.^10^ Blood urea nitrogen, glomerular filtration rate as well as CSF/serum albumin ratio as a measure of BBB permeability were available for all individuals. The presence of KD was defined if the glomerular filtration rate was < 60 mL/min/1.73 m^2^.^11^ With respect to CSF/serum albumin ratio, patients were categorized into those with low, medium, and high ratio based on the tertile partition of the cohort as previously described by Bellaver et al.^12^ Detailed information about how patients with AD, PD, PD with dementia (PDD), DLB, and FTD were diagnosed is present in the Supplementary Material. The UNIPG cohort was used to determine the distribution of plasma Aβ42/40 and p‐tau181 in the different A/T categories and clinical groups, to evaluate the impact of KD and BBB permeability on the concordance between CSF and plasma biomarkers, and for model training.

#### 2.1.2 AMS cohort

The cohort included 80 individuals and was composed of *n* = 40 patients with AD dementia (AD‐dem) from the Amsterdam Dementia Cohort,^13^, ^14^ and of *n* = 40 cognitively healthy control (CTRL) participants from the Dutch Brain Research Registry (Hersenonderzoek.nl).^15^ Diagnosis of AD‐dem was based on the NIA‐AA diagnostic criteria,^3^, ^13^ and confirmed using previously measured CSF Aβ42, CSF t‐tau, and CSF p‐tau181 Elecsys assays analyzed on the Cobas e 601 analyzer (Roche Diagnostics GmbH, Penzberg, Germany). For the CTRL group, no brain amyloid PET or CSF data were available. The *AMS* cohort was used for validation of the results.

### 2.2 Fluid biomarker measurements

We assessed the analytical performance (sensitivity, precision, parallelism, recovery and dilution linearity) of the Lumipulse assays using pooled remnant, anonymous EDTA plasma samples at the Neurochemistry Laboratory, Amsterdam UMC, according to the method developed by the BIOMARKAPD consortium.^16^ Acceptance criteria were set as a %CV of less than 20% for precision and %‐agreement calculations falling within the range of 80%–120% for the remaining analytical validation parameters (see Table S2).

For both cohorts, the plasma samples were collected from all participants in dipotassium EDTA anticoagulant tubes through venipuncture. After a 10 min centrifugation at 1800–2000 × *g*, plasma was aliquoted in 0.5mL portions in polypropylene storage tubes (Sarstedt, Germany) and stored at −80°C until use. The plasma samples were shortly thawed at room temperature (15°C to 25°C) for 30 min, vortexed for 10 s, and centrifuged for 5 min at 2000 × g just before the measurements. The Aβ40, Aβ42, and p‐tau181 measurements were performed using fully automated CLEIA on the LUMIPULSE G System (LUMIPULSE G600II) according to the instructions from the manufacturer at the University of Perugia, Perugia, Italy, or at Amsterdam UMC, Amsterdam, The Netherlands. In all clinical and analytical validation measurements we used the diluents supplied with the CLEIA kits according to the instructions of the manufacturer. For the *UNIPG cohort*, CSF and plasma Aβ40, Aβ42, and p‐tau181 were measured in singlicate. For the *AMS cohort*, plasma Aβ40 and Aβ42 were measured in singlicate and plasma p‐tau181 was measured in duplicate. Detailed information about the assays is mentioned in Supplementary Material.

### 2.3 Statistical analysis

Differences in age among CSF A/T groups were assessed by means of Dunn's test with Benjamini–Hochberg (BH) correction to account for false discovery rate (FDR). Differences in sex prevalence among the same groups were assessed by the chi‐square test with BH correction. The influence of KD on plasma biomarkers was assessed by means of Mann–Whitney U‐test, in the UNIPG cohort. Passing‐Bablok regressions and Spearman's correlation analysis were applied to understand the impact of BBB permeability on the agreement between plasma and CSF biomarkers in the UNIPG cohort. The statistical significance of the differences among correlations was assessed by using the “cocor” R package.^17^

**RESEARCH IN CONTEXT**

1. **Systematic review**: The literature was reviewed using conventional sources (e.g., Pubmed and Google Scholar). The current literature suggests that plasma biomarkers hold promise for introduction into the clinic for noninvasive molecular diagnosis of Alzheimer's disease (AD). Their measurement by automated platforms may further fill the gaps needed for their clinical application.
2. **Interpretation**: High plasma p‐tau181 accurately mirrors CSF A+/T+ levels with strong sensitivity and specificity in dementia and mild cognitive impairment (MCI) stages, but less effectively in preclinical AD, where plasma Aβ42/40 ratio performs better. Low Aβ42/40 consistently indicates A+ status regardless of T and clinical condition. Assays exhibit consistent, reliable performance, supporting cost‐effective, accessible blood‐based biomarker assessment using automated platforms.
3. **Future directions**: Employment of probabilistic algorithms may allow the use of plasma p‐tau181 and Aβ42/40 in the clinics, especially at MCI and dementia stage where their performance is comparable to cerebrospinal fluid analysis.

For assessment of the differences in plasma biomarkers among the four different CSF A/T groups in the UNIPG cohort, logistic regression was used for each pairwise comparison by considering age and sex as covariates. For each pairwise comparison, effect size was also calculated by means of Cohen's d. ROC analysis was performed to assess the diagnostic potential of plasma biomarkers in differentiating among CSF A/T categories and clinical groups using the R packages pROC^18^ and AUROC (for age‐adjusted receiver operating characteristic [ROC] curves).^19^ Confidence intervals for sensitivity, specificity, and area under the ROC curve (AUC) were computed by generating 2000 bootstrap replicates. The bootstrap method was also used to assess the statistical significance for differences in (partial) AUCs of unpaired ROC curves. The Venkatraman method^20^ was used to compare paired ROC curves.

To determine cutoff points and classification models, we used probability density function fitting in a way similar to that applied by Gobom et al. for the determination of CSF Aβ42/40 cutoff value.^21^ Probability density fitting for plasma Aβ42/40 and plasma p‐tau181 was performed by applying the “fitdist” function of the “fitdistrplus” R package.^22^ The goodness of fit was assessed by comparison of empirical and estimated histograms and cumulative density functions (CDF), quartile‐quartile (Q‐Q) plots, and probability‐probability (P‐P) plots. From fitted univariate probability densities, the cutoff value for plasma Aβ42/40 was determined at *p* = 0.5 for the A+ versus A‐ comparison, while that of p‐tau181 was determined at *p* = 0.5 for the A+/T+ versus non‐A+/T+ comparison. Cutoff values for *p* = 0.1, 0.3, 0.7, and 0.9 were also reported. Bivariate classification models (making use of both plasma p‐tau181 and Aβ42/40) were computed by a weighted average of probability values previously determined by univariate probability densities. The optimal combination constant, for each model, was chosen by grid search by maximizing model accuracies by means of 100‐fold cross‐validation in the training set (*UNIPG* cohort). These constants (k = 0.663 for A+ versus A‐ model and k = 0.167 for the A+/T+ versus not A+/T+ model) were then used to perform a weighted average of probabilities resulting from univariate plasma Aβ42/40 and plasma p‐tau181 fitted probability densities. For bivariate models predicting A+ and A+/T+ CSF profiles, isoprobability curves are reported for *p* = 0.1, 0.3, 0.5, 0.7, and 0.9. For all the classification models (univariate and bivariate), sensitivities, specificities, and accuracies have been calculated in the whole training set (*UNIPG* cohort, A+ vs. A‐ and A+/T+ vs. not A+/T+), in subsets of the training set (all AD vs. CTRL, preAD vs. CTRL, MCI‐AD vs. CTRL, and AD‐dem vs. CTRL), and in the external validation cohort (*AMS* cohort, AD‐dem vs. CTRL). A trinomial classification algorithm was also applied to predict CSF A/T profile from bivariate models. The algorithm consisted in the serial application of the A+/T+ versus not A+/T+ and the A+ versus A‐ binomial bivariate models and allowed the detection of the A+/T+, A+/T‐, and A‐/T ± profiles from plasma Aβ42/40 and p‐tau181 data. The R code to apply all the developed models, trained on *UNIPG* data, is present in the Supplementary Material.

## 3 RESULTS

### 3.1 Study participants

#### 3.1.1 UNIPG cohort

Number of included subjects, age, sex, and values of measured plasma biomarkers of the included patients are reported in Table 1 grouped by both CSF A/T and clinical classification. When grouped on A/T classification, the number of subjects with KD and the percentage of subjects belonging to each CSF/serum albumin tertiles was also reported. Among the CSF A/T categories, subjects with CSF A‐/T‐ (67.6 ± 8.1 y) and A‐/T+ profiles (69.6 ± 6.7 y) were younger than those with CSF A+/T‐ (72.1 ± 5.6 y, *p* < 0.001 and *p* = 0.01, respectively) and CSF A+/T+ profiles (72.3 ± 6.1 y, *p* < 0.001 for both). The A+/T+ groups also had a higher percentage of female subjects (64.5%) than the A‐/T‐ (43.6%, *p* = 0.0005) and A‐/T+ (40.0%, *p* = 0.0008) groups. Mean age was greater in all AD patients (72.4 ± 6.0 y) than in CTRL (67.9 ± 8.7 y, *p* = 0.0002).

**TABLE 1:** Demographic details, plasma biomarker values, and prevalences of A/T CSF profiles of the *UNIPG* and *AMS* cohorts.

*UNIPG* cohort											
CSF A/T groups	N (450 total)	Sex (M/F)	Age (y)	KD+/‐	CSF/serum Alb. high	CSF/serum Alb. medium	CSF/serum Alb. low	Plasma Aβ40 (pg/mL)	Plasma Aβ42 (pg/mL)	Plasma Aβ42/40	Plasma p‐tau181 (pg/mL)
A‐/T‐	126	71/55	67.6 ± 8.1	11/115	29%	34%	37%	301 ± 76	25.7 ± 7.3	0.085 ± 0.012	1.59 ± 1.3
A‐/T+	50	30/20	69.6 ± 6.7	1/49	29%	29%	42%	312 ± 95	26.1 ± 7.1	0.085 ± 0.016	1.68 ± 0.73
A+/T‐	48	23/25	72.1 ± 5.6	1/47	32%	41%	27%	319 ± 63	22.5 ± 5.5	0.071 ± 0.012	1.77 ± 0.72
A+/T+	226	80/146	72.3 ± 6.1	15/211	36%	32%	32%	303 ± 56	22.4 ± 5.3	0.074 ± 0.009	2.92 ± 1.3

Clinical groups	N (450 total)	Sex (M/F)	Age (y)	A‐/T‐	A‐/T+	A+/T‐	A+/T+	Plasma Aβ40 (pg/mL)	Plasma Aβ42 (pg/mL)	Plasma Aβ42/40	Plasma p‐tau181 (pg/mL)
CTRL‐CN	46	29/17	65.2 ± 9.4	100%	0%	0%	0%	291 ± 53	25.5 ± 4.9	0.088 ± 0.011	1.59 ± 1.50
CTRL‐MCI	38	18/20	71.3 ± 6.5	100%	0%	0%	0%	331 ± 114	27.5 ± 11	0.082 ± 0.016	1.75 ± 1.53
PD‐CN	20	12/08	66.6 ± 7.0	55%	35%	5%	5%	289 ± 47	25.7 ± 6.4	0.089 ± 0.012	1.28 ± 0.62
PD‐MCI	25	15/10	67.9 ± 6.4	52%	16%	20%	12%	284 ± 77	22.8 ± 4.4	0.082 ± 0.011	1.55 ± 0.71
PDD/DLB	15	13/02	71.7 ± 7.6	27%	0%	27%	47%	312 ± 45	23.3 ± 4.9	0.075 ± 0.011	2.45 ± 0.92
preAD	31	11/20	71.6 ± 5.8	0%	0%	0%	100%	302 ± 42	23.1 ± 3.5	0.077 ± 0.008	1.91 ± 0.76
MCI‐AD	104	31/73	72.1 ± 5.5	0%	0%	0%	100%	303 ± 58	22.2 ± 6.3	0.073 ± 0.011	2.93 ± 1.50
AD‐dem	81	27/54	73.0 ± 6.7	0%	0%	0%	100%	307 ± 58	22.7 ± 4.6	0.074 ± 0.007	3.36 ± 0.98
FTD	31	17/14	69.7 ± 6.3	42%	29%	29%	0%	322 ± 105	25.6 ± 6.9	0.081 ± 0.011	1.84 ± 0.77
other	59	31/28	70.9 ± 6.5	0%	47%	53%	0%	306 ± 56	23.4 ± 6.2	0.077 ± 0.017	1.63 ± 0.64

*AMS* cohort											
Clinical groups	*N* (80 total)	Sex (M/F)	Age (y)	A‐/T‐	A‐/T+	A+/T‐	A+/T+	Plasma Aβ40 (pg/mL)	Plasma Aβ42 (pg/mL)	Plasma Aβ42/40	Plasma p‐tau181 (pg/mL)
HC	40	12/28	65.0 ± 6.7	–	–	–	–	299 ± 50	27.1 ± 5.01	0.088 ± 0.014	1.82 ± 1.07
AD‐dem	40	12/28	65.2 ± 9.4	0%	0%	0%	100%	292 ± 32	22.1 ± 2.50	0.076 ± 0.007	3.64 ± 0.99

*Note*: *UNIPG* cohort is described considering both cerebrospinal fluid (CSF) A/T classification and clinical classification (upper and lower part of the table, respectively). Continuous variables are represented as mean ± standard deviation. Sex and kidney dysfunction prevalences are described as n° of males/n° of females and n° KD+/n° KD‐, respectively. CSF/serum albumin (Alb.) tertiles prevalences within the A/T groups and CSF A/T profiles prevalences within clinical groups are represented as percentages. The University of Perugia (*UNIPG)* cohort consists of cognitively normal subjects with A‐/T‐ CSF profiles (CTRL‐CN), subjects with mild cognitive impairment due to non‐neurodegenerative diseases (e.g., vascular, polyfactorial, CTRL‐MCI) with A‐/T‐ CSF profile, cognitively normal subjects affected by Parkinson's disease (PD‐CN), subjects with mild cognitive impairment associated to Parkinson's disease (PD‐MCI), subjects with Parkinson's disease with dementia (PDD) or dementia with Lewy bodies (DLB), cognitively normal subjects with Alzheimer's disease‐like CSF profile (preAD), subjects with mild cognitive impairment due to Alzheimer's disease (MCI‐AD), subjects with Alzheimer's disease dementia (AD‐dem), and subjects with frontotemporal dementia (FTD). The “other” group consists of subject selected to populate the A+/T‐ and A‐/T+ categories, that is, subjects with suspected non‐Alzheimer's pathology (A‐/T+ CSF profile) and subjects with isolated cerebral amyloidosis (A+/T). The Amsterdam (*AMS)* cohort was composed of AD‐dem subjects with A+/T+ CSF profile and age/sex‐matched healthy controls with unknown CSF profile.

Abbreviations: DLB, Lewy body dementia; HC, healthy controls.

Positivity of both amyloid and tauopathy CSF biomarkers (CSF A+/T+) in PD patients increased with disease stage, being 5% in cognitively normal PD and 12% in PD‐MCI. PDD/DLB cases had a CSF A+/T+ profile in 47% of cases, in agreement with previous findings.^23^, ^24^, ^25^ Regarding FTD patients, 42% had CSF A‐/T‐ and the remaining 58% were equally divided between CSF A‐/T+ and A+/T‐.

#### 3.1.2 AMS cohort

AD‐dem patients (*n* = 40) had a mean age of 65 ± 4.2 years, and 28 individuals (70%) were females. CTRL subjects (*n* = 40) had a mean age of 65 ± 6.7 years, and 28 individuals (70%) were females (see Table 1).

### 3.2 Analytical performance of plasma Aβ40, Aβ42, and p‐tau181 assays

The characteristics of the Lumipulse assays are listed in Table S1. Aβ40, Aβ42, and p‐tau181 assays showed good precision, with average intra‐assay coefficients of variation (CV) from duplicate measurements of three quality control samples of 1.9% for Aβ40, 3.8% for Aβ42, and 3.3 % for p‐tau181, and average inter‐assay CVs of the three samples measured over 3 days of 9.1 % for Aβ40, 5.6 % for Aβ42, and 10.4 % for p‐tau181. In agreement, using *UNIPG cohort plasma QCs*, we obtained average intra‐assay CVs of 3.1 % for Aβ40, 2.1 % for Aβ42, and 3.3 % for p‐tau181, and average inter‐assay CVs of 5.2 % for Aβ40, 8.0% for Aβ42, and 5.4 % for p‐tau181. Likewise, for the measurements performed in AMS, duplicate measurements for p‐tau181 in the *n* = 80 *AMS* samples showed an average CV of duplicate measurements of 2.1%, and below 20%CV for all samples (Figure S1 in Supplementary Material; Aβ40 and Aβ42 assays were run in singlicates in the clinical samples). The lowest limit of quantification (LLoQ) of the assays were 1.9 pg/mL for the Aβ40, 0.75 pg/mL for the Aβ42, and 0.47 pg/mL for the p‐tau181. The assays showed excellent parallelism of values measured in four times two‐fold diluted plasma samples compared to the assays calibration curves, with slopes close to 100% (mean parallelism response of 104% with Aβ40, 98% with Aβ42, and 93% with p‐tau181 assays); high average recoveries of low, medium, and high‐concentrated spikes with the calibrator proteins in samples (109% with Aβ40, 91% with Aβ42, and 89% with p‐tau181 assays); and good dilution linearity responses of samples that were spiked with calibrator proteins to reach concentrations above the standard curve and subsequently serially diluted until below the LLoQ (97% over six dilution points between 1‐ and 1024‐fold with Aβ40, 91% for six dilution points over the range of 1‐ to 1024‐fold with Aβ42, and 111% over the range of 1‐ to 256‐fold dilutions with p‐tau181 assays).

### 3.3 Influence of KD and BBB permeability on plasma Aβ40, Aβ42, and p‐tau181

In the *UNIPG* cohort, to analyze the impact of KD on plasma biomarkers, patients were solely stratified into A+ and A‐ according to CSF values of Aβ42/40, in order to maintain an acceptable sample size. Within the A‐ group, KD+ patients showed significantly higher values of plasma Aβ40 (Cohen's d = 0.76, *p* = 0.000098), Aβ42 (Cohen's d = 0.84, *p* = 0.0061), p‐tau181 (Cohen's d = 1.84, *p* = 0.00002) compared to KD‐ patients, while plasma Aβ42/40 showed comparable values between the two groups (Cohen's d = 0.15, *p* = 0.12) (Figure S2 in Supplementary Material). We computed the ratios p‐tau181/Aβ40 (Cohen's d = 0.36, *p* = 0.022), and p‐tau181/Aβ42 (Cohen's d = 1.18, *p* = 0.0038), but they did not fully compensate the effect of KD on p‐tau181. Within the A+ group, on the contrary, KD+ and KD‐ patients showed no significant differences in each of the plasma biomarkers and ratios measured (Figure S2 in Supplementary Material).

Following previous findings,^12^ in order to determine the influence of BBB permeability, we stratified patients and controls from the *UNIPG* cohort into those with low, medium, and high CSF/serum albumin ratio. We found the highest agreement between CSF and plasma Aβ42/40 in the “high” CSF/serum albumin ratio tertile (*ρ* = 0.666), while significantly weaker correlations were found in the “medium” and “low” tertiles (*ρ* = 0.403, *p* comparison = 0.008 and *ρ* = 0.474, *p* comparison = 0.012, respectively). Not statistically significant differences among correlations were found for p‐tau181 (*ρ* = 0.677 in “high” tertile, *ρ* = 0.621 for “medium” and *ρ* = 0.655 for “low”) (Figure S3 in Supplementary Material).

### 3.4 Plasma Aβ42/40 and p‐tau181 across CSF A/T categories

**FIGURE 1:**
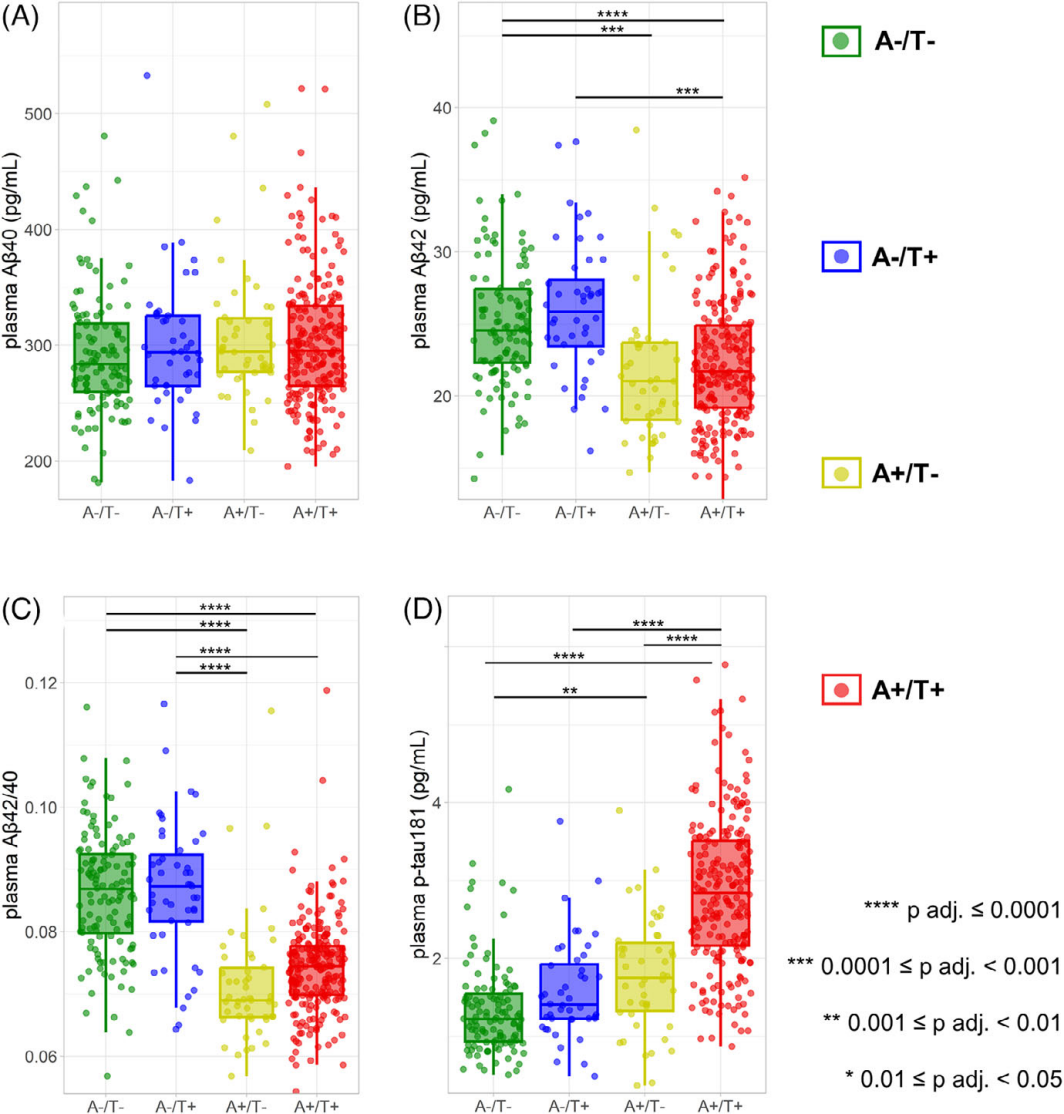
Plasma Alzheimer's disease (AD) biomarker concentrations in the four cerebrospinal fluid (CSF) A/T categories. Plasma amyloid β (Aβ)40 (A), Aβ42 (B), Aβ42/40 (C), and p‐tau181 (D) concentrations in subjects with A‐/T‐, A‐/T+, A+/T‐, and A+/T+ cerebrospinal fluid (CSF) profiles are displayed as boxplots in which the boxes represent the interquartile range, the horizontal lines within boxes represent the median concentrations, and whiskers reflect the first/third quartile ‐/+ 1.5 times the interquartile range. The *p*‐values reported are calculated by logistic regression for pairwise comparisons and are adjusted for age and false discovery rate.

We have analyzed plasma biomarker levels in the four CSF A/T categories. Boxplots relative to plasma levels of Aβ40, Aβ42, Aβ42/40, and p‐tau181 and *p*‐values adjusted for age and sex differences are shown in Figure 1, and additional boxplots for p‐tau181/Aβ40 and p‐tau181/Aβ42 are shown in Figure S4 (Supplementary Material). Effect‐sizes are shown for each pairwise comparison among CSF A/T groups in Table S2 (Supplementary Material).

Plasma levels of Aβ42 and Aβ42/40 were significantly lower in A+ patients, independently from T status, compared to A‐ patients. Plasma p‐tau181 levels were significantly higher in A+/T+ subjects compared to all the other groups. We could not detect any significant alteration in any of the biomarkers considered in the A‐/T+ group as compared to A‐/T‐, including plasma p‐tau181. Significant but small, in terms of effect size (Table S2), differences were found in p‐tau181 between the CSF A‐/T‐ and A+/T‐ groups.

**FIGURE 2:**
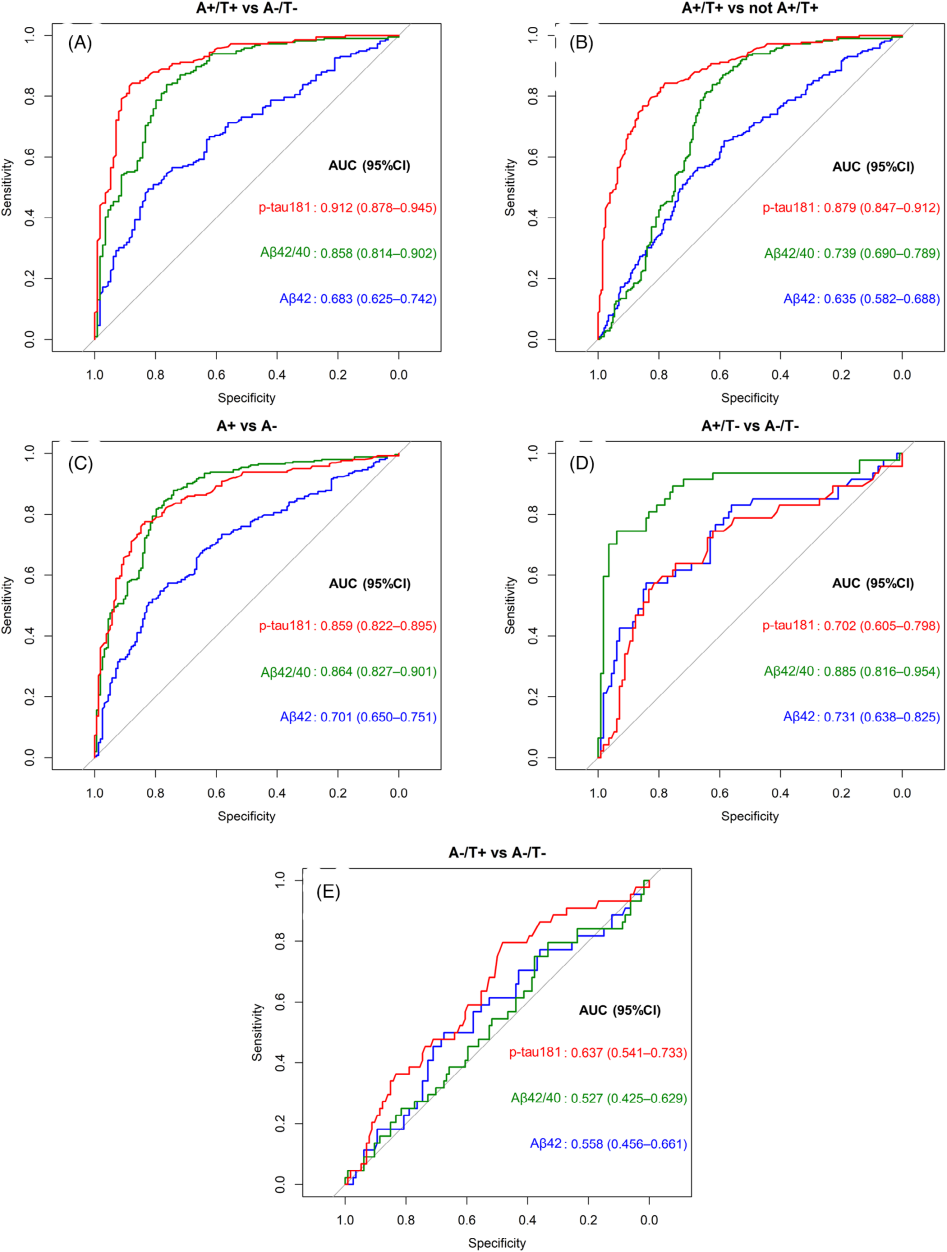
Receiver operating characteristic (ROC) curves quantifying the ability of plasma amyloid β (Aβ)42, Aβ42/40, and p‐tau181 in differentiating among cerebrospinal fluid (CSF) A/T profiles. Areas under the ROC curves (AUC) are displayed for each comparison together with their 95% confidence interval calculated by generating 2000 bootstrap replicates. Plasma p‐tau181 exhibited high diagnostic performance in detecting the A+/T+ versus A‐/T‐ (A) and vs all the other profiles (B). Plasma Aβ42/40 demonstrated a high performance in detecting the A+ profile versus A‐ (C), being also able to detect the A+/T‐ profile versus A‐/T‐ (D). None of the plasma biomarkers considered was able to detect the A‐/T+ CSF profile (E).

The observed differences for the plasma biomarkers and biomarker ratios were then confirmed by ROC analysis (Figure 2). Among all the biomarkers and ratios investigated, plasma p‐tau181 showed the best performance in the comparisons A+/T+ versus A‐/T‐, and A+/T+ versus all the other categories (not A+/T+). Plasma Aβ42/40 showed the best performance in the comparisons A+ versus A‐ and A+/T‐ versus A‐/T‐. In all comparisons, performances of Aβ42 alone were significantly lower compared to Aβ42/40. AUC values adjusted for age differences among groups (Table S2) were similar to unadjusted AUC values (Figure 2). None of the considered biomarkers or their ratios were able to distinguish between the CSF A‐/T‐ and A‐/T+ groups. ROC analysis for p‐tau181/Aβ40 and p‐tau181/Aβ42 is also reported in Figure S5 (Supplementary Material). Computing these two ratios did not result in a significant improvement of the diagnostic performance as compared to p‐tau181 and Aβ42/40.

The comparisons for which plasma biomarkers showed the highest specificity, that is, A+/T+ versus all the other groups together, and A+/T+ versus A‐/T‐ for plasma p‐tau181, A+ versus A‐ and A+/T+ versus A‐/T‐ for plasma Aβ42/40, were repeated by subgrouping patients according to CSF/serum albumin ratio tertiles (Figure S6 in Supplementary Material). In these comparisons, the performance of plasma p‐tau181 was almost identical in all the CSF/serum albumin ratio tertiles, while for Aβ42/40 the highest AUCs were found in the high tertile, but no statistically significant differences among AUCs were observed.

### 3.5 Plasma Aβ42/40 and p‐tau181 across clinical groups and clinical stages of AD

Plasma Aβ42/40 and p‐tau181 values are summarized for all the clinical groups of the *UNIPG* cohort in Table 1. For each pairwise comparison, *p*‐value ranges adjusted for FDR are present in Table S3 (Supplementary Material).

Plasma p‐tau181 and Aβ42/40 showed no differences between CTRL‐CN and CTRL‐MCI, supporting the choice of referring these two categories as CTRL for the purposes of this analysis.

**FIGURE 3:**
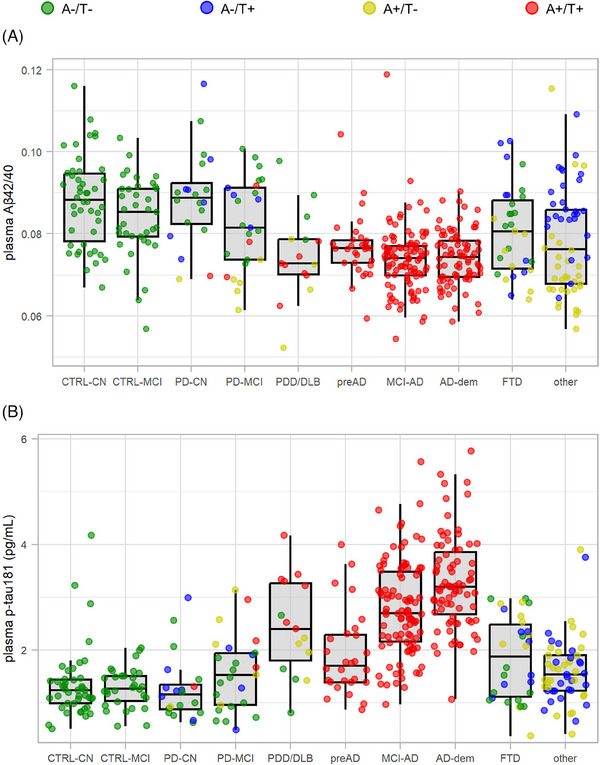
Plasma p‐tau181 and amyloid β (Aβ)42/40 concentrations in clinical groups. Plasma Aβ42/40 (A) and p‐tau181 (B) concentrations are displayed as boxplots for each of the clinical groups considered. Boxes width represent the interquartile range, the horizontal lines within boxes represent the median concentrations, and whiskers reflect the first/third quartile ‐/+ 1.5 times the interquartile range. For each box subjects with A‐/T‐, A‐/T+, A+/T‐, and A+/T+ cerebrospinal fluid (CSF) profiles are highlighted in green, blue, yellow, and red, respectively.

Plasma Aβ42/40 exhibited the lowest values in AD‐dem patients (0.074 ± 0.007) and MCI‐AD patients (0.073 ± 0.011), followed by the PDD/DLB group (0.075 ± 0.011) and preAD group (0.077 ± 0.008). Plasma Aβ42/40 was specifically associated to CSF A status, independently from clinical groups (Figure 3A).

AD‐dem patients exhibited the highest concentrations of plasma p‐tau181 (3.36 ± 0.98 pg/mL), followed by MCI‐AD (2.93 ± 1.5 pg/mL) and PDD/DLB (2.45 ± 0.92). CTRL‐CN and CTRL‐MCI had the lowest concentrations of plasma p‐tau181 (1.59 ± 1.5 and 1.75 ± 1.53 pg/mL, respectively). The preAD group demonstrated significantly lower mean plasma p‐tau181 level compared to MCI‐AD and AD‐dem (1.91 ± 0.76 pg/mL vs. 2.93 ± 1.5 and 3.36 ± 0.98, p‐adj. = 0.011 and 0.0097, respectively) (Table 1) (Figure 3B). For each AD stage, we computed *p*‐values adjusted for age, sex, and FDR and effect sizes (Table S4 in Supplementary Material).

**FIGURE 4:**
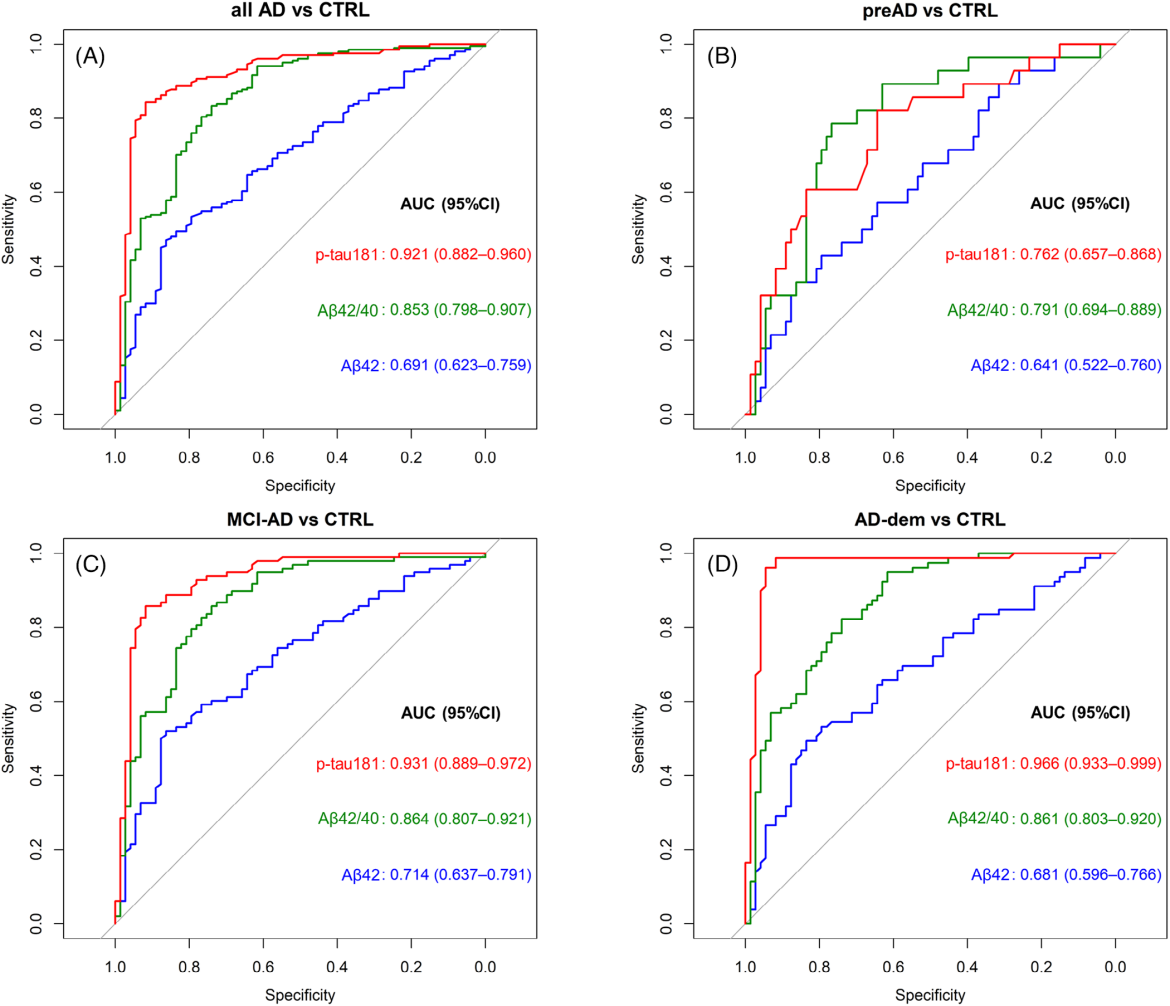
Receiver operating characteristic (ROC) curves quantifying the ability of plasma Aβ42, Aβ42/40, and p‐tau181 in differentiating clinical Alzheimer's disease (AD) stages from healthy controls (CTRL). Areas under the ROC curves (AUC) are displayed for each comparison together with their 95% confidence interval calculated by generating 2000 bootstrap replicates. Plasma p‐tau181 exhibited high diagnostic performance in detecting all AD cases (A), MCI‐AD (C), and AD‐dem (D) versus CTRL. Plasma Aβ42/40 produced the best performance in detecting preAD versus CTRL (B).

ROC curves for plasma Aβ42, Aβ42/40, and p‐tau181 were then computed for all AD patients as well as for each AD clinical stage (preAD, MCI‐AD, and AD‐dem) versus CTRL (Figure 4). Plasma p‐tau181 provided the highest AUCs (> 0.90), except for preAD versus CTRL (AUC = 0.76). In this category, plasma Aβ42/40 provided a higher AUC (0.79), although the difference with p‐tau181 was not statistically significant. In each comparison, Aβ42/40 showed invariably higher accuracy with respect to Aβ42 alone. Comparable AUC values for each plasma biomarker were obtained after accounting for age differences among the clinical groups (Table S4). Additional ROC curves for p‐tau181/Aβ40 and p‐tau181/Aβ42 are displayed in Figure S7 (Supplementary Material). These two ratios provided AUCs equivalent to those of plasma p‐tau181 alone.

### 3.6 Development and testing of probabilistic classification models

We utilized probability density fitting to determine cutoff points, to build classification models, and to determine classification probability for the clinical use of plasma Aβ42/40 and p‐tau181. In training cohort (*UNIPG*), the distribution of plasma Aβ42/40 values in both A‐ and A+ groups were effectively approximated by normal distributions (Figure S8 in Supplementary Material). On the other hand, the empirical plasma p‐tau181 distributions in A+/T+ and non‐A+/T+ groups were best approximated by gamma distributions (Figure S8).

**FIGURE 5:**
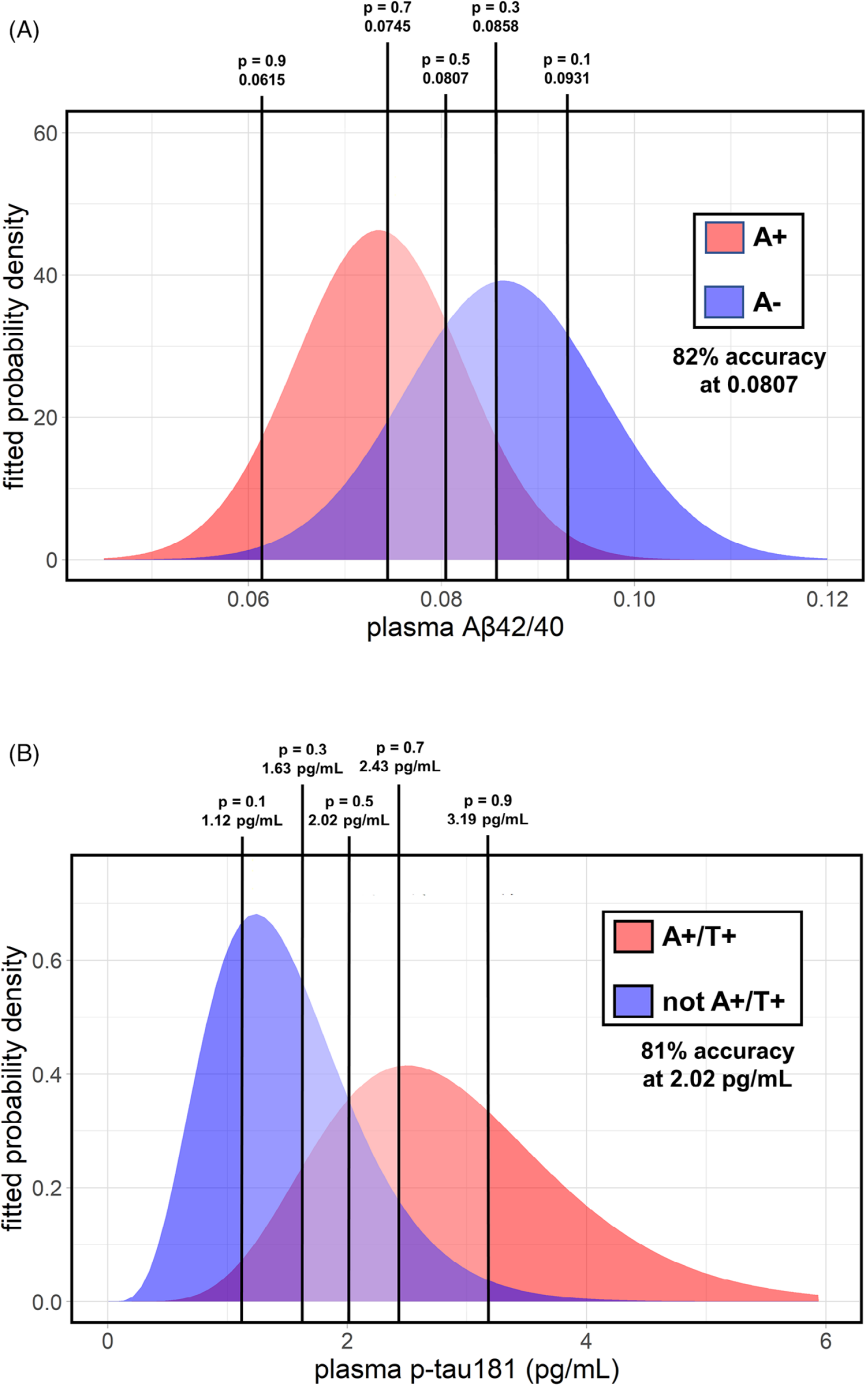
Univariate fitted distributions of plasma amyloid β (Aβ)42/40 and p‐tau181 for A+ versus A‐ and for A+/T+ versus not A+/T+, respectively. Plasma Aβ42/40 data (A) were fitted by means of normal distributions while plasma p‐tau181 (B) by means of gamma distributions. Plasma biomarker levels corresponding to probabilities of 0.1, 0.3, 0.5, 0.7, and 0.9 of being classified as A+ and A+/T+ are reported for Aβ42/40 and p‐tau181, respectively. The classification accuracy at *p* = 0.5 is also shown for the two univariate models.

The fitted probability density functions were then used for classification and to compute cutoff points relative to probabilities of 0.1, 0.3, 0.5, 0.7, and 0.9 of being classified as A+ versus A‐ based on plasma Aβ42/40 and as A+/T+ versus not A+/T+ based on plasma p‐tau181 (Figure 5). The model based on plasma Aβ42/40 for predicting A+ yielded an overall accuracy of 82%, while the model based on plasma ptau181 for predicting A+/T+ yielded an accuracy of 81%. The cutoff values determined by applying these models were 0.0807 for Aβ42/40 (A+ vs. A‐) and 2.02 pg/mL for p‐tau181 (A+/T+ vs. not A+/T+). Computing cutoff values by ROC analysis (by maximizing the Youden index) would have resulted in very similar values, that is, 0.0800 (95 % confidence interval [CI]: 0.0784‐0.0817) for Aβ42/40 and 2.08 (95% CI: 1.92–2.27) pg/mL for p‐tau181 for the same comparisons.

**FIGURE 6:**
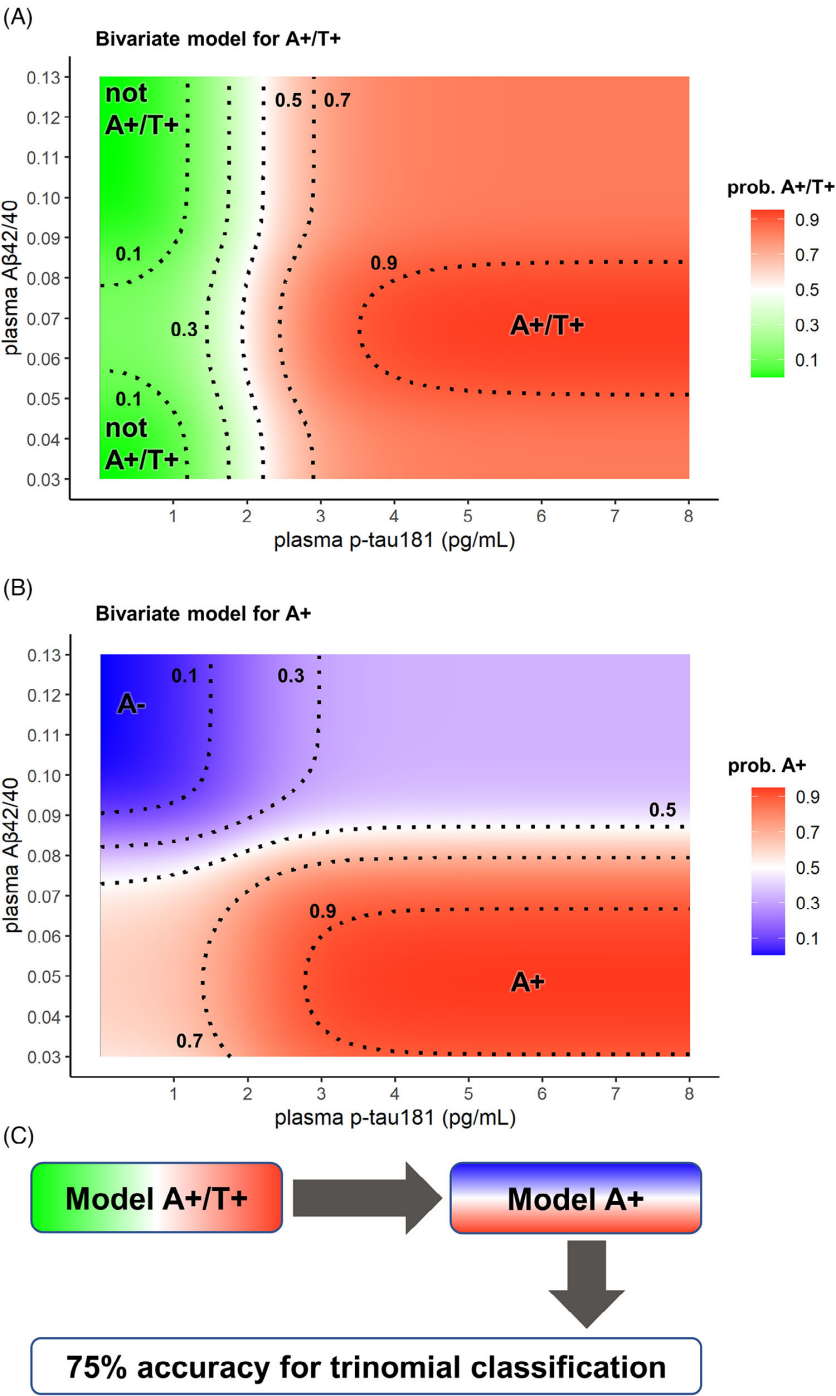
Bivariate probability models. The 2D probability plots for A+/T+ versus not A+/T+ (A) and for A+ versus A‐ (B) were built by combining 1D probabilities calculated for plasma Aβ42/40 and p‐tau181. The optimal combination constant for each of the two models was chosen by maximizing the average accuracy on the training set by 100‐fold cross‐validation. Isoprobability dotted lines for *p* = 0.1, 0.3, 0.5, 0.5, and 0.9 are displayed. (C) Suggested way to combine the two bivariate models for trinomial classification.

Plasma Aβ42/40 and p‐tau181 fitted probability densities were then combined to produce bivariate models (Figure 6). To combine them, plasma Aβ42/40 values were fitted with normal distributions in both the A+/T+ and not A+/T+ groups and plasma p‐tau181 concentrations were fitted with gamma distributions in the A+ and A‐ groups (Figure S9 in Supplementary Material).

In both the univariate and bivariate models, for binomial classifications, we calculated sensitivity, specificity, and accuracy in the training cohort (*UNIPG* cohort), as well as in subsets of the training cohort representing different clinical stages of AD and CTRL. Additionally, we performed these calculations in the *AMS* cohort, which served as an external validation cohort. We considered both raw classification outcomes and outcomes excluding results with uncertain classification, that is, as an example, within the 0.3–0.7 probability range. The results of these analyses are shown in Table 2. With reference to Table 2, the univariate A+ model (considering only plasma Aβ42/40) was able to reflect CSF A state with similar performance (sensitivity: 90%–82%, specificity: 69%–73%, accuracy: 76%–82%) in all comparisons considered. Notably, the results obtained in the external validation cohort (composed of AD‐dem and CTRL patients) were almost identical to those obtained in the training cohort for AD‐dem versus CTRL. In contrast, the univ. A+/T+ model (based solely on plasma p‐tau181) showed near‐optimal performance in detecting AD‐dem in the training (sensitivity: 96%) and validation (sensitivity: 98%) cohorts, high performance in detecting MCI‐AD in the training cohort (sensitivity: 80%), and low performance in detecting pre‐AD in the training cohort (sensitivity: 32%). The overall specificity of plasma p‐tau181 in the training cohort was 93% in CTRL subjects and 79% in not A+/T+ subjects, while in the validation cohort, for controls with unknown CSF status, it was 80%. Bivariate models combining plasma Aβ42/40 and p‐tau181 for A+ and A+/T+ classification generally performed better than univariate models. Because the bivariate A+/T+ model was more dependent on plasma p‐tau181 than Aβ42/40, it had very‐high diagnostic performance for the MCI‐AD and AD‐dem in both cohorts (accuracy: 86%–96%), while it showed poor sensitivity in detecting preAD (36%). The bivariate A+ model had almost identical performance for A+ versus A‐, MCI‐AD versus CTRL, AD‐dem versus CTRL in the training cohort and for AD‐dem versus CTRL in the validation cohort (sensitivity: 92%–94%, specificity: 77%–78%, accuracy: 85%–87%) and lower performance in differentiating preAD from CTRL (sensitivity: 71%, specificity: 77%, accuracy: 75%). Our approach allowed us to obtain classification probabilities; thus, we were able to determine the variation in classification accuracy by excluding classifications made within certain probability ranges. For example, by excluding classifications with probabilities in the range 0.3–0.7, we could observe an overall increase in sensitivity, specificity, and accuracy in each of the classifications made. The least evident increase (from 73%–80% to 81%–84%) was that obtained in the specificities measured in the validation cohort (AMS) for which the CSF and renal function status of the control subjects were not available. Finally, we tried to explore the maximum potential of our models. By applying the two binomial bivariate models in series (Figure 6C), it was possible to perform trinomial classification for identifying A+/T+, A+/T‐, and A‐/T ± CSF profiles. This approach led to an overall 75% trinomial accuracy in the *UNIPG* cohort (see “Trin.” in Table 2 and confusion matrices in Table S5). The procedure of applying the two bivariate models in series was repeated, by excluding classifications with probabilities within the 0.3–0.7 range, leading to an overall 91% trinomial accuracy in the *UNIPG* cohort.

**TABLE 2:** Summary of the diagnostic performance of the probabilistic classification models.

	Sensitivity				Specificity				Accuracy					
	univ. A+	univ. A+/T+	biv. A+	biv. A+/T+	univ. A+	univ. A+/T+	biv. A+	biv. A+/T+	univ. A+	univ. A+/T+	biv. A+	biv. A+/T+	biv. trin.	
*UNIPG* cohort	88%	80%	90%	83%	73%	79%	77%	80%	82%	80%	85%	82%	75%	
all AD versus CTRL	87%	80%	90%	83%	69%	93%	77%	93%	82%	83%	87%	86%	–	Color scale
preAD versus CTRL	82%	32%	71%	36%	69%	93%	77%	93%	76%	76%	75%	77%	–	100%
MCI‐AD versus CTRL	90%	80%	94%	85%	69%	93%	77%	93%	81%	86%	87%	88%	–	90%
AD‐dem versus CTRL	85%	96%	92%	99%	69%	93%	77%	93%	77%	95%	85%	96%	–	80%
*AMS* cohort	83%	98%	93%	98%	73%	80%	78%	80%	78%	89%	85%	89%	–	70%
														60%

	Sensitivity at prob. > 0.7 or prob. < 0.3				Specificity at prob. > 0.7 or prob. < 0.3				Accuracy at prob. > 0.7 or prob. < 0.3					50%
	univ. A+	univ. A+/T+	biv. A+	biv. A+/T+	univ. A+	univ. A+/T+	biv. A+	biv. A+/T+	univ. A+	univ. A+/T+	biv. A+	biv. A+/T+	biv. trin.	40%
*UNIPG* cohort	90%	82%	94%	87%	82%	87%	92%	87%	87%	86%	93%	87%	91%	30%
all AD versus CTRL	90%	82%	96%	86%	87%	95%	93%	95%	89%	88%	95%	89%	–	20%
preAD versus CTRL	82%	56%	75%	29%	87%	95%	93%	95%	84%	81%	91%	81%	–	10%
MCI‐AD versus CTRL	92%	79%	95%	89%	87%	95%	93%	95%	90%	90%	94%	92%	–	
AD‐dem versus CTRL	92%	88%	100%	99%	87%	95%	93%	95%	89%	97%	97%	97%	–	
*AMS* cohort	92%	100%	96%	100%	84%	82%	83%	81%	87%	90%	89%	90%	–	

*Note*: Sensitivity, specificity, and accuracy were calculated from the univariate models based on plasma Aβ42/40 (univ. A+) and p‐tau181 (univ. A+/T+) and for bivariate binomial models based on both plasma Aβ42/40 and p‐tau181 (biv. A+ and biv. A+/T+) on the training cohort (*UNIPG* cohort), in subsets of the *UNIPG* cohort consisting of CTRL and all AD, preAD, MCI‐AD, and AD‐dem, and on the external validation cohort (*AMS* cohort). The trinomial prediction accuracy (number of correct predictions out of the total number of samples) for the bivariate trinomial model is also reported. Model performances were then recalculated by considering solely outcomes with probabilities greater than 0.7 or smaller than 0.3 (robust outcomes).

Abbreviations: Univ, univariate binomial; biv, bivariate; trin, Trinomial.

## 4 DISCUSSION

In the present study, we investigated whether plasma levels of Aβ42, Aβ42/40, and p‐tau181, measured by CLEIA on a fully automated random‐access platform, accurately reflect CSF A/T profiles and clinical AD classification across the AD clinical spectrum, as well as in comparison to other neurodegenerative conditions. Two independent cohorts were considered, one for model training, and the other one for model testing.

Our main findings can be summarized in four main points:

- All the tested assays demonstrated an excellent analytical performance.
- High plasma p‐tau181 accurately reflects CSF A+/T+ profile in AD‐dem and MCI‐AD. In preclinical AD the sensitivity of plasma p‐tau181 was unsatisfactory.
- Low Aβ42/40 plasma consistently reflected A+ status, irrespective of T status and clinical stage. Plasma Aβ42 alone exhibited a significantly lower performance compared to the Aβ42/40.
- We developed classification algorithms defining probability of patient's A+/T+ or A+ CSF profile based on measurement of plasma Aβ42/40 and p‐tau181.

In this study, we observed an excellent analytical performance of the plasma Aβ40, Aβ42, and p‐tau181 CLEIA assays. The measurements of the QC panels exhibited high consistency and low variability, with an inter‐assay coefficient of variation (CV) ≤ 10%. This high precision, attributed to the automated systems utilized (i.e., Lumipulse G), enhances the reproducibility of blood biomarker measurements. The assays also demonstrated good sensitivity, as none of the samples fell below the LLoQ for any of the biomarkers considered. Furthermore, the plasma assays exhibited good parallelism and recovery responses, with mean responses falling within the accepted ranges. Dilution linearity assessment revealed that the Lumipulse assays displayed a good linearity across a wide range of concentrations, suggesting a reliable measurement without saturation or dilution effects.

In terms of concordance with CSF A/T profile, increased plasma p‐tau181 was confirmed to be a reliable indicator of CSF A+/T+ profile, showing AUCs of 0.879 (0.847‐0.912) for A+/T+ versus not A+/T+ and 0.912 (0.878‐0.945) for A+/T+ versus A‐/T‐. Plasma p‐tau181 levels were significantly elevated in patients showing CSF A+/T+ profiles compared to all other A/T categories, including A‐/T+. Indeed, plasma p‐tau181 could not identify CSF A‐/T+ profile. This profile, which generally accounts for less than 5% of cases,^26^, ^27^ was recently found not to be associated with higher rates of cognitive deterioration, Aβ accumulation, tau PET pathology, or neurodegeneration with respect to A‐/T‐ CSF profile.^26^ Consequently, the lack of identification of the A‐/T+ CSF profile may not be clinically significant, as this profile does not seem to reflect a specific ongoing pathophysiology in the central nervous system (CNS) but rather nonpathological aspects, such as altered CSF turnover. Therefore, it has recently been proposed that, in clinical practice, an A‐/T+ CSF profile should be considered normal, similar to an A‐/T‐ profile.^26^ Thus, our results confirm that plasma p‐tau181 could serve as surrogate biomarker of overall AD pathology rather than solely reflecting CSF p‐tau181 status, in agreement with the literature.^2^

With respect to clinical diagnoses, plasma p‐tau181 showed a noticeable trend of increasing values along the AD continuum, being higher in more advanced clinical stages (AD‐dem > MCI‐AD > preAD). Therefore, the best diagnostic accuracy of plasma p‐tau181 in discriminating AD patients from controls was observed at dementia and MCI stages. In the category of preclinical AD, sensitivity of elevated p‐tau181 concentrations was poor, and plasma Aβ42/40 achieved a better sensitivity in discriminating preAD patients from controls.

Another interesting aspect is the ability of plasma Aβ42/40 to detect A+ status, independently from clinical group considered and disease stage. In a previous study assessing performance of eight different assays for plasma detection Aβ42/40, AUCs ranging from 0.64 to 0.78, were obtained for discrimination between A+ and A‐ subjects.^28^ In our series, Lumipulse measurement of plasma Aβ42/40 enabled a better discrimination between A+ and A‐ patients, that is, AUC of 0.864 (0.827–0.901).

We found also that only plasma Aβ42/40 (differently from Aβ42, Aβ40, p‐tau181, p‐tau181/Aβ42, and p‐tau181/Aβ40) was the biomarker which levels did not differ between subjects with normal and impaired renal function, irrespective of A status. This finding further strengthens the use of plasma Aβ42/40, as it is not impacted by potential confounding factors like kidney diseases/dysfunction. On the contrary, impaired renal function will likely generate false positive results on plasma p‐tau181.^11^

Following the results obtained by Bellaver et al.,^12^ we considered BBB permeability (reflected by CSF/serum albumin ratio levels) as another potential confounder. The highest agreement between CSF and plasma biomarkers – both Aβ42/40 and p‐tau181 – was observed in patients with the highest CSF/serum albumin ratio. The difference between the highest CSF/serum albumin ratio tertile and the lower ones was statistically significant for Aβ42/40, which supports the influence of BBB permeability on the concordance between specific CSF and plasma biomarkers.^12^

Finally, we calculated probability profiles where specific values of plasma markers corresponded to predefined A/T categories in CSF. We assessed the probability that a certain value of plasma Aβ42/40 corresponded to CSF A+. Similarly, we examined the probability that a specific value of plasma p‐tau181 corresponded to an A+T+ categorization in CSF. In these models, plasma Aβ42/40 < 0.0807 and plasma p‐tau181 > 2.02 pg/mL were identified as the cutoff points for predicting CSF A+ and A+/T+ profiles, respectively. In the bivariate analyses, we combined the measurements of these two biomarkers to determine the probability of CSF A+/T+ and A+ profiles. For example, having a plasma Aβ42/40 = 0.07 and p‐tau181 = 3.5 pg/mL confers a 90% probability of having a CSF A+T+ profile. These two bivariate models can be utilized to generate the probability of CSF A+ or A+/T+ CSF profiles from plasma data or can be applied in series for trinomial classification, that is, by allowing the identification of A+/T+, A+/T‐, and A‐/T ± CSF profiles. For instance, if the plasma Aβ42/40 is 0.065 and p‐tau181 is 1.5 pg/mL, there is a 31% probability of having a CSF A+T+ profile and a 70% probability of having a CSF A+ profile, thus suggesting an A+/T‐ profile. All binomial models were evaluated for sensitivity, specificity, and accuracy in different clinical stages of AD and CTRL subsets in the *UNIPG* cohort and validated using the *AMS* cohort. The performance of all models for A+/T+ classification improved with the progression of AD clinical stages, yielding accuracies greater than 95% in AD patients with dementia compared with controls. The trinomial accuracy resulting from the serial application of the A+/T+ and A+ bivariate models in the whole *UNIPG* cohort was also satisfactory (75%). In summary, our bivariate models have been shown to be applicable to assess the presence of AD in subjects with MCI and dementia (after excluding the presence of renal dysfunction).

The main strengths of our study are: (i) the use of a large cohort comprising 450 paired CSF/plasma samples (*UNIPG* cohort), (ii) the inclusion of an external validation cohort (*AMS* cohort), (iii) the harmonization of the preanalytical variables across the two cohorts, (iv) the inclusion of a consistent number of samples belonging to subjects with infrequent CSF A/T profiles (A+/T‐ and A‐/T+), (iv) the inclusion of renal function and BBB permeability data, and (v) the use of probability densities, which has enabled us to develop models that overcome the constraints of linear decision lines commonly observed in traditional logistic models and offer classification probabilities, an attribute that is not easily obtained by alternative methods such as decision trees.

With respect to limitations, first, although significant alterations in plasma markers were observed when accounting for renal dysfunction, the limited number of KD+ subjects constrained our ability to incorporate this feature into models or to further explore compensatory strategies. Second, the absence of data on renal function and BBB permeability for subjects in the validation cohort, as well as the lack of CSF profiles for the 40 control subjects, suggests that the model specificities calculated on the *AMS* cohort may have been underestimated. Last, the evaluation of performance of our classification models in identifying preAD was constrained by the modest size of this group. Furthermore, the lack of preclinical subjects in the validation cohort indicates that the ability of plasma p‐tau181 and Aβ42/40 in detecting preAD must be further assessed in larger, independent cohorts.

Overall, our results confirm the robust analytical performance of plasma AD biomarkers, also in fully automated assays. Since blood‐based AD diagnosis offers advantages in terms of accessibility and repeatability, plasma biomarkers have great potential for routine clinical use, as well as for recruitment and monitoring in clinical trials. In the future, it will be mandatory to further investigate plasma/CSF concordance in preclinical AD stages, as well as to better quantify the impact of KD and other systemic comorbidities on plasma AD biomarkers measurements.

## CONFLICT OF INTEREST STATEMENT

Prof. Parnetti served as member of advisory boards for Fujirebio, I.B.L., Roche, and Merck and has a collaboration contract with ADx, Amprion Inc., and Fujirebio. Prof Chalotte Teunissen has a collaboration contract with ADx Neurosciences, Quanterix, Eli Lilly, and Fujirebio. Prof. Wiesje Flier served as a member of advisory boards of Biogen, Roche, and Eli Lilly and is recipient of ABOARD, which is a public‐private partnership receiving funding from ZonMW (#73305095007). All the other authors report no conflicts of interest. Author disclosures are available in the supporting information.

## CONSENT STATEMENT

All the procedures involving human subjects were performed following Helsinki Declaration. All participants gave written informed consent to use medical data and biomaterials for research purposes. The study was approved by the local Ethics Committees (Comitato Etico Aziende Sanitarie Regione Umbria 19369/AV and 20942/21/OV; Amsterdam UMC VUmc medical ethical committee 2019.102).

## Supporting information

Supporting Information

Supporting Information

## Data Availability

The data that support the findings of this study are available upon reasonable request from the corresponding author. Researchers interested in accessing the data can contact Prof. Lucilla Parnetti at lucilla.parnetti@unipg.it to initiate the data sharing process. The R code needed to apply the classification models developed in this study is present in the last paragraph of the Supplementary Material file and can be tested by readers on their own data.
