# Supplementary material for: Fully automated measurement of plasma Aβ42/40 and p‐tau181: Analytical robustness and concordance with cerebrospinal fluid profile along the Alzheimer's disease continuum in two independent cohorts
Source: Alzheimers Dement. 2024 Feb 7;20(4):2453–68. doi: 10.1002/alz.13687 (PMC11032583; doi:10.1002/alz.13687)
Supplement: Supplementary file 1 — Supporting Information [file ALZ-20-2453-s001.docx]

**Supplementary material**

**Study participants: the *UNIPG* cohort**

All AD patients were diagnosed according to the 2018 NIA-AA criteria [1], showing at least a CSF A+/T+ profile. Clinical stage of AD patients was defined according to the neuropsychological assessment and Clinical Dementia Rating (CDR) scale. Patients with Parkinson’s disease (PD), PD with dementia (PDD) and dementia with Lewy bodies (DLB) were diagnosed according to the current diagnostic criteria [2–4]. Based on neuropsychological assessment, PD patients were categorized as cognitively normal PD (PD-CN) and PD with MCI (PD-MCI) patients according to the criteria of Litvan et al. of 2012 [5]. Considering the limited number of PDD and DLB subjects, we decided to consider these groups together (PDD/DLB). The diagnosis of frontotemporal dementia (FTD) was made on a clinical basis supported by ^18^F-FDG-PET [6,7]. As clinical control group, we considered cognitively healthy subjects (CTRL-CN) and subjects with MCI not due to AD or PD (CTRL-MCI), i.e., vascular MCI, for whom neurodegenerative disorders were excluded. All CTRL subjects showed a CSF A-/T- profile. Considering the need of populating the A-/T+ and A+/T- CSF profile categories, which usually accounts for ~5% of the cases [8], we also included individuals with suspected non-AD pathology, and subjects with isolated cerebral amyloidosis (CSF A+/T- profile). In all these cases, PD, PDD/DLB and FTD were excluded, and these patients were grouped under the label “other”.

**Plasma Aβ40, Aβ42 and p-tau181 assays**

The plasma Aβ40 and Aβ42 Lumipulse® assays utilize a two-step setup where the analytes are captured on 2G3-coated or 21F12-coated beads, respectively, and then detected with ALP-labelled 3D6 conjugates after washing. The Aβ40 and Aβ42 assays employ peptides as the calibrators. The plasma p-tau181 Lumipulse® assay utilizes a two-step setup where the analyte is initially captured on AT270 coated beads and then detected with ALP-labelled HT7 / BT2 conjugate after washing. The assay employs a synthetic peptide containing the three epitopes as the calibrator.


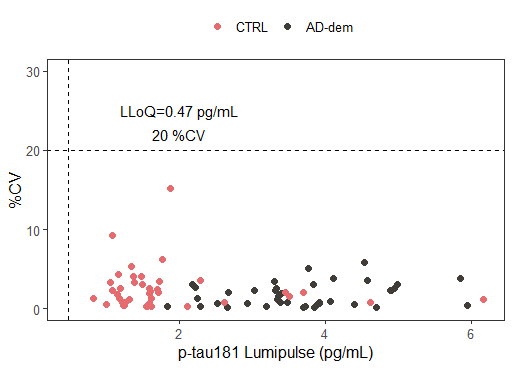


**Figure S1.** **Precision profile of the p-tau181 assay using the n=80 samples of the validation cohort.** The X-axis demonstrate plasma p-tau181 levels measured by the Lumipulse® GII (pg/mL) and the Y-axis shows variability (coefficient of variation: %CV) in duplicate measurements of the clinical samples of the validation cohort. The vertical dashed line was set at the determined lowest limit of quantification (LLoQ) and the horizontal line was set at CV of 20% as the maximum accepted %CV.

|  | | Aβ40 | | Aβ42 | | p-tau181 | |
| --- | --- | --- | --- | --- | --- | --- | --- |
| Assay | Provider | Fujirebio Europe | | Fujirebio Europe | | Fujirebio Europe | |
|  | Status | Commercial  (Research use) | | Commercial  (Research use) | | Commercial  (Research use) | |
|  | Catalogue number | N/A | | N/A | | N/A | |
|  | Biofluid | EDTA plasma | | EDTA plasma | | EDTA plasma | |
| Platform |  | Lumipulse® G 600 | | Lumipulse® G 600 | | Lumipulse® G 600 | |
| Calibration curve | No. of calibrator points | 5 | | 5 | | 5 | |
|  | Range, pg/mL | 0-5000 | | 0-1000 | | 0-60 | |
|  | Curve fit | 1/y²-weighted 4PL | | 1/y²-weighted 4PL | | 1/y²-weighted 4PL | |
| Sample dilution | Fold-dilution | Undiluted | | Undiluted | | Undiluted | |
|  | Recommended method | Fully Automated | | Fully Automated | | Fully Automated | |
| Clinical samples measurements | Number | 80 | | 80 | | 80 | |
|  | Range concentration, pg/mL | 85-424 | | 9.5-38.7 | | 0.81- 6.17 | |
|  | Range, CV% | Singlicate | | Singlicate | | 0.0-15.24 | |
|  | Average CV% | Singlicate | | Singlicate | | 2.1 | |
|  | n measured <LLoQ | 0 | | 0 | | 0 | |
|  | n measured >20%CV | NA | | NA | | 0 | |
| Analytical validation results | | | | | | | |
| Sensitivity | Analytical LLoQ, pg/mL | 1.91 | | 0.75 | | 0.47 | |
| Concentrations of QC panels | QC1: high, pg/mL | NA | | 10.3 | | 12 | |
|  | QC2: intermediate, pg/mL | 212 | | 11.4 | | 4.7 | |
|  | QC3: low, pg/mL | 163 | | 12.6 | | 1.5 | |
| Precision of QCs | Average Intra-assay %CV | 2.3 | | 3.8 | | 3.3 | |
|  | Average Inter-assay %CV | 9.1 | | 5.6 | | 10.4 | |
| Parallelism | Average slope of samples | 0.94 | | 0.90 | | 0.84 | |
|  | Range of slopes of samples | 0.94-1.0 | | 0.79-0.93 | | 0.79-0.89 | |
|  | Average slope of calibrator | 0.94 | | 0.93 | | 0.90 | |
|  | Parallelism, % | 104 | | 98.0 | | 93.0 | |
| Dilution linearity | Spiked concentration, pg/ml | 1841 | | 205 | | 248 | |
|  |  | Df (x) | Mean %L | Df (x) | Mean %L | Df (x) | Mean %L |
|  | Linear dilution factor  with mean %Linearity | 1 | - | 1 | - | 1 | - |
|  |  | 4 | 92 | 4 | 92 | 4 | 92 |
|  |  | 16 | 87 | 16 | 86 | 16 | 87 |
|  |  | 64 | 85 | 64 | 92 | 64 | 85 |
|  |  | 256 | 97 | 256 | 91 | 256 | 97 |
|  |  | 1024 | 123 | 1024 | 95 | 1024 | 123 |
| Recovery | Spiked concentration (pg/mL)  With mean %Recovery | Spike | Mean %R | Spike | Mean %R | Spike | Mean %R |
|  |  | 200 | 106.24 | 25 | 88.9 | 3.2 | 91 |
|  |  | 400 | 108.87 | 50 | 89.8 | 28.3 | 94 |
|  |  | 800 | 110.72 | 100 | 94.1 | 53.9 | 83 |

**Table S1. Analytical characteristics of the plasma Lumipulse® G assays.** Analytical LLoQ was calculated as the mean signal of 16 blanks plus 10 times the SD, with the p-tau concentration extrapolated from the calibration curve. This was multiplied by the sample dilution factor to obtain the functional LLOD. QC samples are EDTA plasma pools, and specific to each assay. Average intra-and inter-assay variation was derived from measuring the QC panels over four independent runs. For the p-tau181 assay, 80 clinical samples were measured in duplicate, while for the Aβ40 and Aβ42 assays were measured only in singlet. For parallelism, with each assay, four samples were measured after being four-times 2-fold serially diluted (p-tau181, starting dilution 2-fold reaching 16-fold). For dilution linearity, three samples were spiked with high recombinant protein concentration, subsequently measured undiluted and serially diluted until low p-tau, Aβ40 or Aβ42 concentrations below the LLoQs of the assays. p-tau=phosphorylated tau; PL=polynomial; LLoQ=lower limit of quantification; QC=quality control; CV=coefficient of variation; %L=% linearity; %R=% recovery; NA=not applicable.


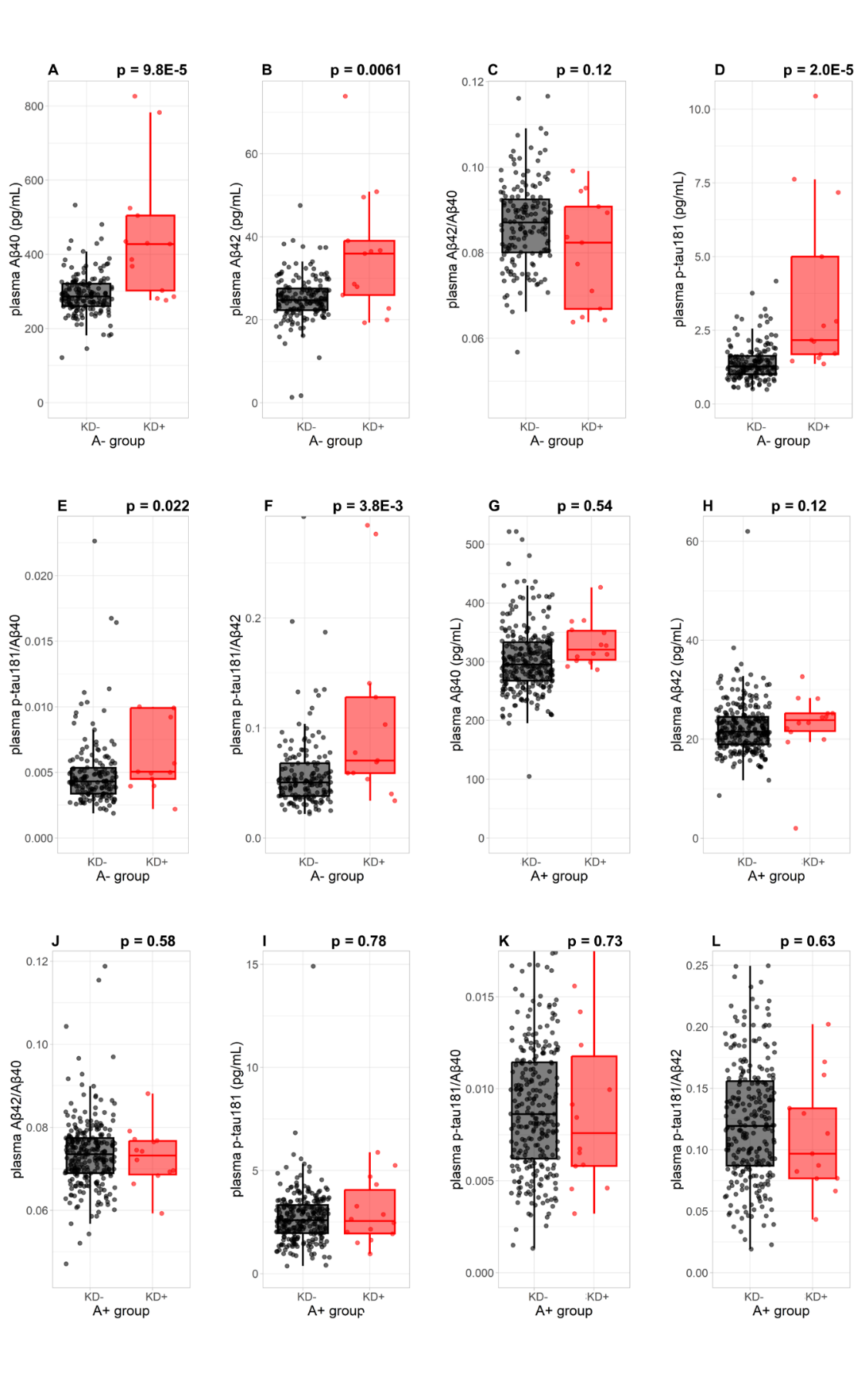


**Figure S2. Plasma AD biomarker concentrations and their ratios in CSF A+/A- categories for subjects with and without KD.** Plasma biomarker concentrations in KD- and KD+ subjects are displayed as boxplots in which the boxes represent the interquartile range, the horizontal lines within boxes represent the median concentrations and whiskers reflect the first/third quartile -/+ 1.5 times the interquartile range.


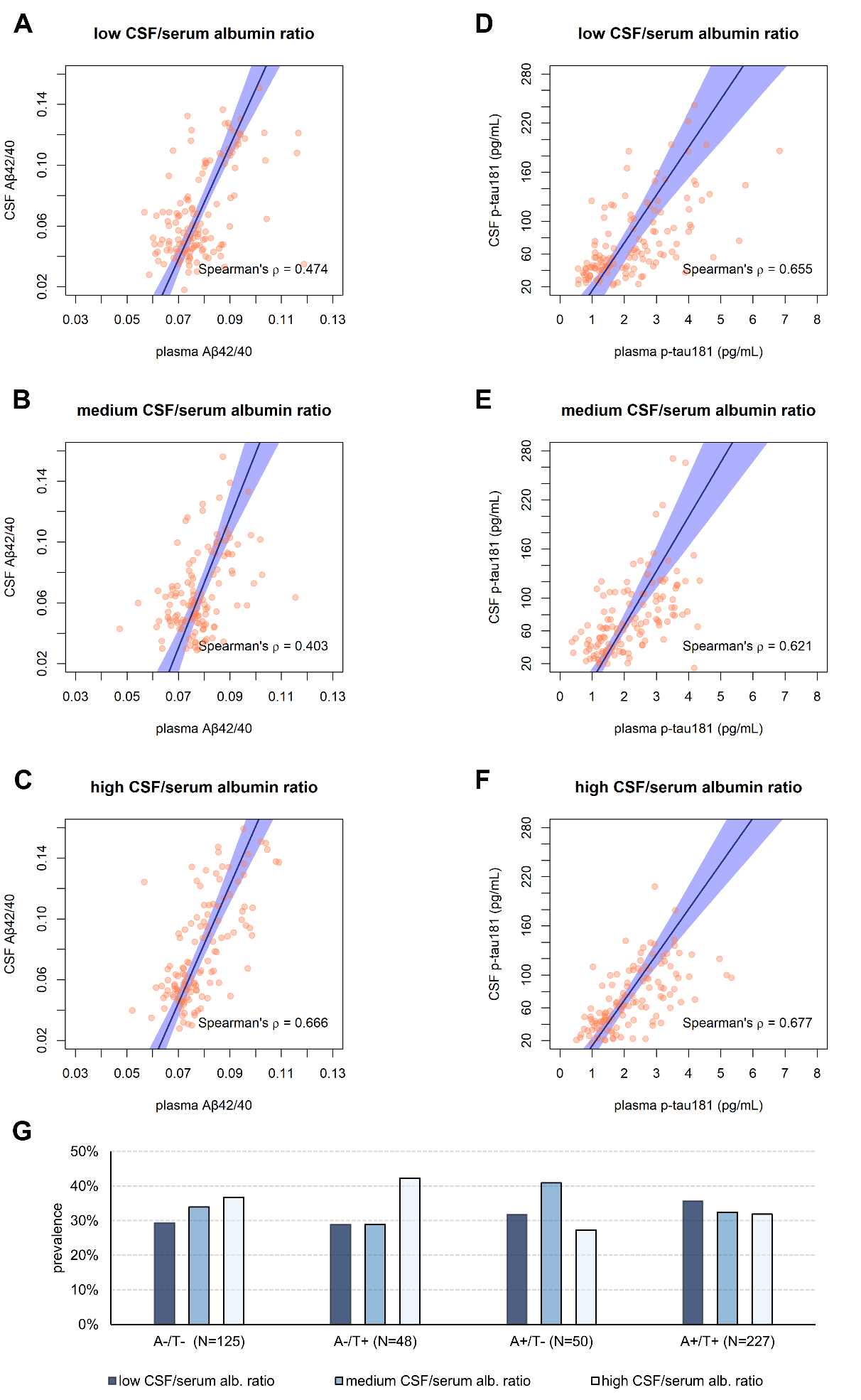


**Figure S3. Associations between CSF and plasma Aβ42/40 and p-tau181 in different CSF/serum albumin ratio tertiles.** Passing-Bablok regression and Spearman’s correlation coefficient (ρ) in the ‘low’, ‘medium’ and ‘high’ tertiles for Aβ42/40 (A-C) and p-tau181 (D-F). G) Prevalences of the CSF/serum albumin ratio tertiles in the four CSF A/T categories considered.

| comparison | | plasma Aβ40 | | | plasma Aβ42 | | | plasma Aβ42/Aβ40 | | | plasma p-tau181 | | |
| --- | --- | --- | --- | --- | --- | --- | --- | --- | --- | --- | --- | --- | --- |
| group1 | group2 | d | age/sex adj. p | age adj. AUC | d | age/sex adj. p | age adj. AUC | d | age/sex adj. p | age adj. AUC | d | age/sex adj. p | age adj. AUC |
| A-/T- | A+/T+ | 0.17 | 1 | 0.528 (0.451-0.602) | 0.56 | 1.7E-06 | 0.725 (0.652-0.788) | 1.43 | 1.7E-15 | 0.830 (0.770-0.880) | 1.42 | 3E-16 | 0.907 (0.869-0.938) |
| A-/T- | A+/T- | 0.27 | 1 | 0.517 (0.411-0.621) | 0.68 | 0.0011 | 0.748 (0.648-0.833) | 1.51 | 3.2E-08 | 0.855 (0.771-0.920) | 0.67 | 0.0014 | 0.677 (0.574-0.774) |
| A-/T- | A-/T+ | 0.06 | 1 | 0.502 (0.401-0.604) | 0.10 | 1 | 0.534 (0.433-0.633) | 0.03 | 1 | 0.555 (0.452-0.651) | 0.38 | 0.098 | 0.618 (0.519-0.706) |
| A+/T+ | A+/T- | 0.10 | 1 | 0.524 (0.438-0.615) | 0.10 | 1 | 0.573 (0.474-0.664) | 0.42 | 0.072 | 0.661 (0.563-0.744) | 0.94 | 2.52E-08 | 0.814 (0.747-0.871) |
| A+/T+ | A-/T+ | 0.10 | 1 | 0.530 (0.430-0.626) | 0.65 | 4.3E-05 | 0.756 (0.675-0.828) | 1.51 | 7.85455E-10 | 0.831 (0.747-0.900) | 1.10 | 5.14E-09 | 0.862 (0.794-0.911) |
| A+/T- | A-/T+ | 0.19 | 1 | 0.528 (0.400-0.659) | 0.77 | 0.0044 | 0.701 (0.575-0.807) | 1.42 | 6.98824E-06 | 0.829 (0.725-0.909) | 0.29 | 0.54 | 0.584 (0.460-0.709) |

**Table S2. Effect size (Cohen’s d), p-values adjusted for age, gender and FDR and age-adjusted AUC for pairwise comparisons among CSF A/T profiles for each of the plasma biomarker measured with Lumipulse® G.**


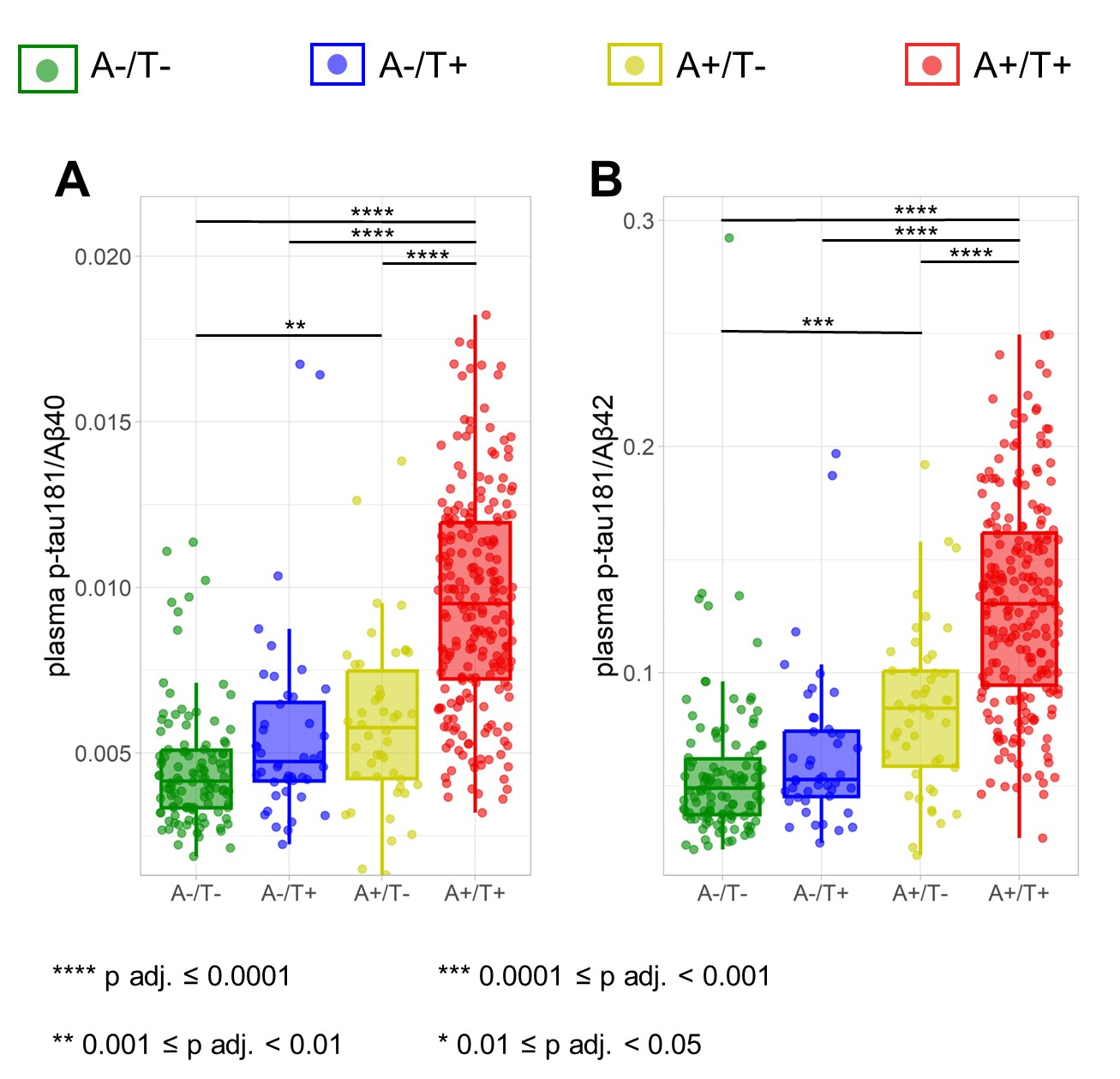


**Figure S4.** Plasma p-tau181/Aβ40 (A) and plasma p-tau181/Aβ42 (B) levels in subjects with A-/T-, A-/T+, A+/T- and A+/T+ CSF profiles are displayed as boxplots in which the boxes represent the interquartile range. The horizontal lines within boxes represent the median concentrations and whiskers reflect the first/third quartile -/+ 1.5 times the interquartile range. The p-values reported are calculated by logistic regression for pairwise comparisons and are adjusted for age and multiple testing effects


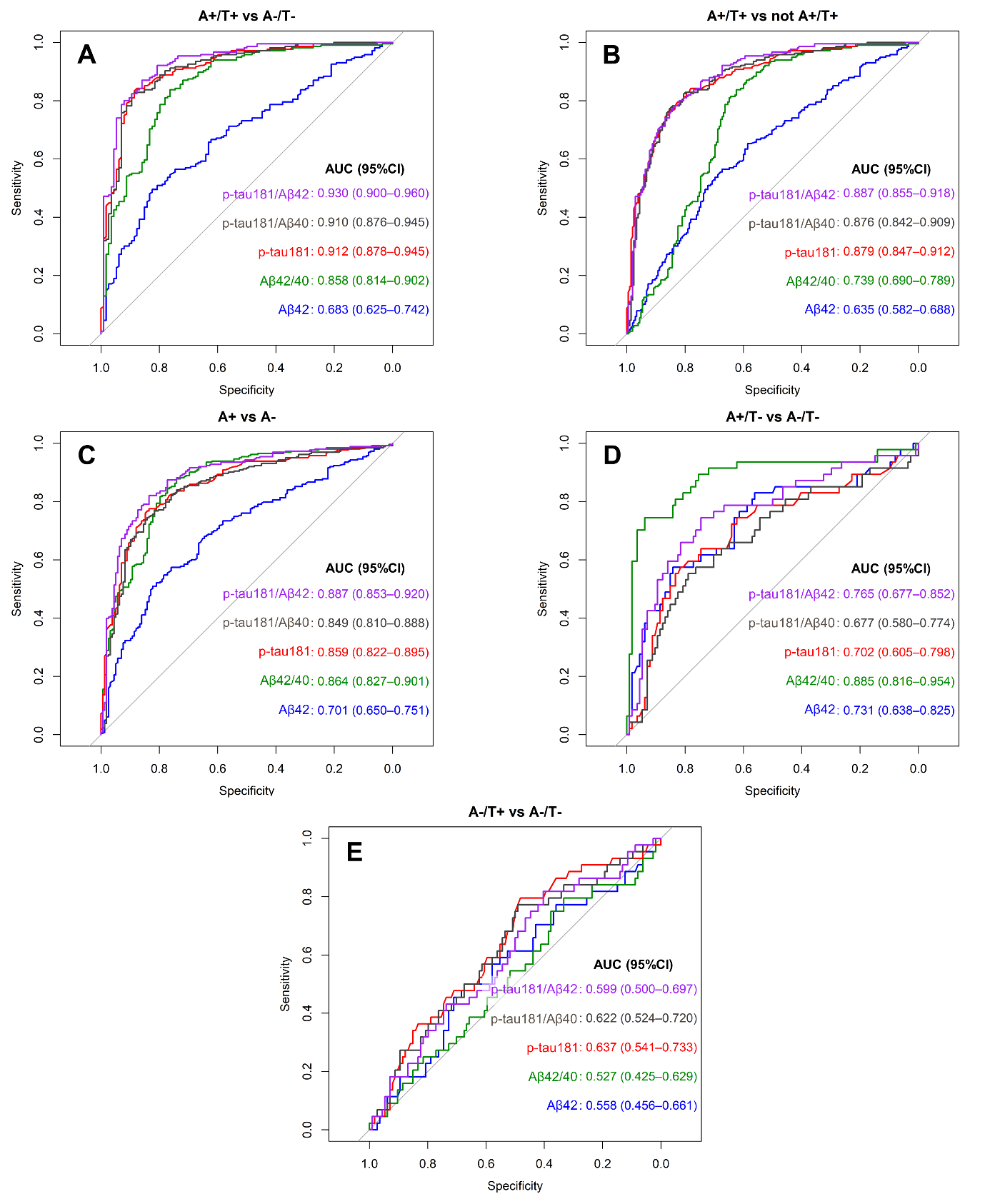


**Figure S5. ROC curves quantifying the ability of plasma Aβ42, Aβ42/40, p-tau181, p-tau181/Aβ40, and p-tau181/Aβ42 in differentiating among CSF A/T profiles.** Areas under the ROC curves (AUC) are displayed for each comparison together with their 95% confidence interval calculated by generating 2000 bootstrap replicates.


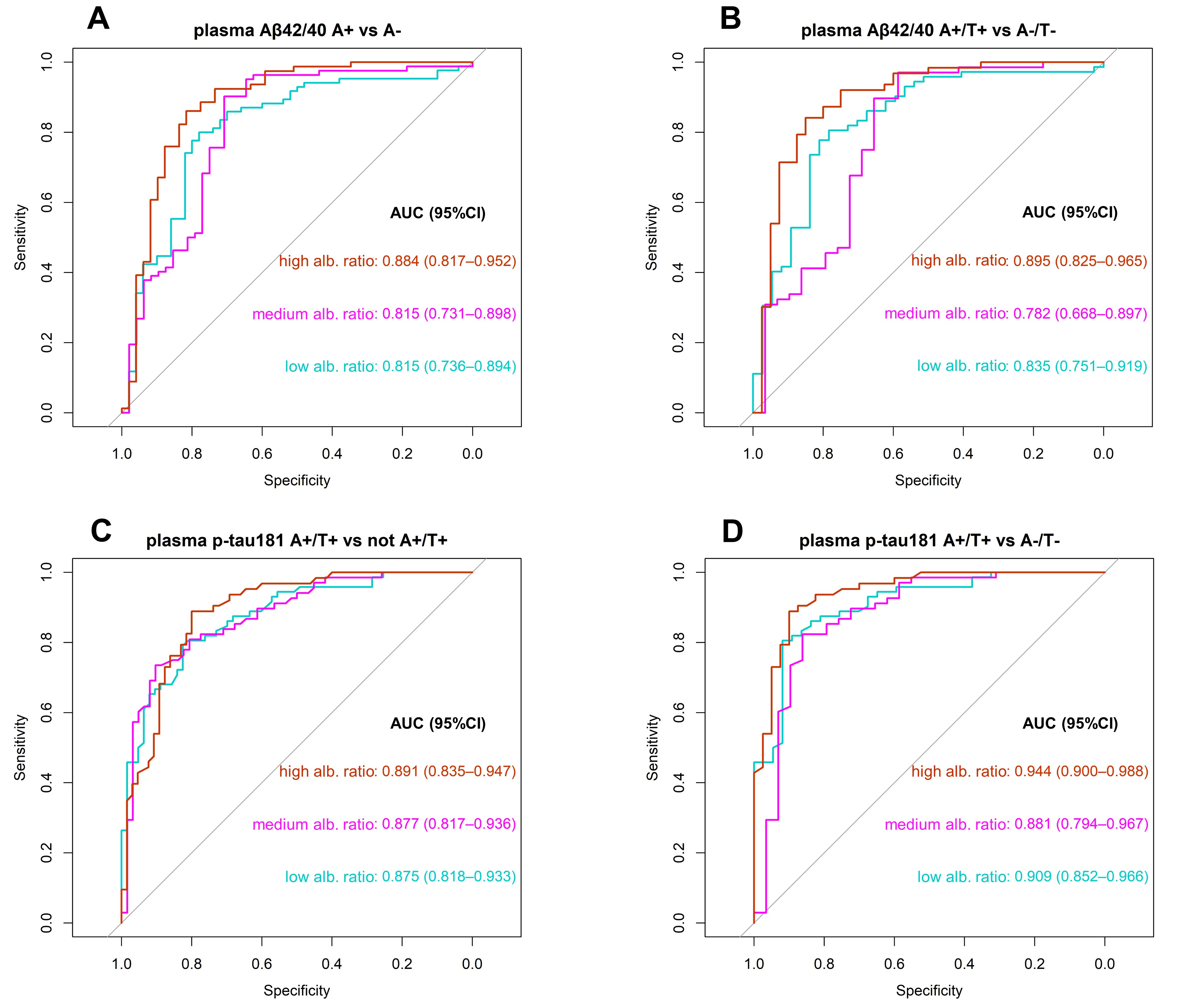


**Figure S6. ROC curves quantifying the effect of BBB permeability on the diagnostic performance of plasma Aβ42/40 and p-tau181.** ROC were calculated in plasma samples belonging to subjects showing low, medium, and high CSF/serum albumin ratio for plasma Aβ42/40 for the A+ vs A- (A) and A+/T+ vs A-/T- comparisons (B) and for plasma p-tau181 for the A+/T+ vs not A+/T+ (C) and A+/T+ vs A-/T- comparisons (D). Areas under the ROC curves (AUC) are displayed for each comparison together with their 95% confidence interval calculated by generating 2000 bootstrap replicates.

| comparison | plasma Aβ40 | plasma Aβ42 | plasma Aβ42/40 | plasma p-tau181 |
| --- | --- | --- | --- | --- |
| AD-dem - CTRL-CN |  | ** | ***** | ***** |
| AD-dem - CTRL-MCI |  | * | ***** | ***** |
| CTRL-CN - CTRL-MCI |  |  |  |  |
| AD-dem - FTD |  |  | ** | ***** |
| CTRL-CN - FTD |  |  | ** | * |
| CTRL-MCI - FTD |  |  |  | * |
| AD-dem - MCI-AD |  |  |  | * |
| CTRL-CN - MCI-AD |  | ** | ***** | ***** |
| CTRL-MCI - MCI-AD |  | ** | ***** | ***** |
| FTD - MCI-AD |  |  | ** | **** |
| AD-dem - other |  |  | * | ***** |
| CTRL-CN - other |  |  | ***** | * |
| CTRL-MCI - other |  |  | ** | * |
| FTD - other |  |  |  |  |
| MCI-AD - other |  |  | * | ***** |
| AD-dem - PD-CN |  |  | ***** | ***** |
| CTRL-CN - PD-CN |  |  |  |  |
| CTRL-MCI - PD-CN |  |  |  |  |
| FTD - PD-CN |  |  | * | * |
| MCI-AD - PD-CN |  | * | ***** | ***** |
| other - PD-CN |  |  | *** | * |
| AD-dem - PD-MCI |  |  | ** | ***** |
| CTRL-CN - PD-MCI |  |  | * |  |
| CTRL-MCI - PD-MCI |  |  |  |  |
| FTD - PD-MCI |  |  |  |  |
| MCI-AD - PD-MCI |  |  | *** | ***** |
| other - PD-MCI |  |  |  |  |
| PD-CN - PD-MCI |  |  |  |  |
| AD-dem - PDD/DLB |  |  |  | * |
| CTRL-CN - PDD/DLB |  |  | *** | *** |
| CTRL-MCI - PDD/DLB |  |  | ** | *** |
| FTD - PDD/DLB |  |  |  | * |
| MCI-AD - PDD/DLB |  |  |  |  |
| other - PDD/DLB |  |  |  | ** |
| PD-CN - PDD/DLB |  |  | ** | *** |
| PD-MCI - PDD/DLB |  |  | * | ** |
| AD-dem - preAD |  |  |  | ***** |
| CTRL-CN - preAD |  |  | *** | ** |
| CTRL-MCI - preAD |  |  | * | ** |
| FTD - preAD |  |  |  |  |
| MCI-AD - preAD |  |  | * | *** |
| other - preAD |  |  |  |  |
| PD-CN - preAD |  |  | ** | ** |
| PD-MCI - preAD |  |  |  |  |
| PDD/DLB - preAD |  |  |  |  |
| * | ** | *** | **** | ***** |
| p adj. ≤ 0.05 | p adj. ≤ 0.01 | p adj. ≤ 0.001 | p adj. ≤ 0.0001 | p adj. ≤ 0.00001 |

**Table S3. P-value ranges (adjusted for FDR), for each pairwise comparison among the clinical groups considered in this study.**

| comparison | | plasma Aβ40 | | | plasma Aβ42 | | | plasma Aβ42/Aβ40 | | | plasma p-tau181 | | |
| --- | --- | --- | --- | --- | --- | --- | --- | --- | --- | --- | --- | --- | --- |
| group1 | group2 | d | age/sex  adj. p | age adj. AUC | d | age/sex adj. p | age adj. AUC | d | age/sex adj. p | age adj. AUC | d | age/sex adj. p | age adj. AUC |
| CTRL | all AD | 0.12 | 1 | 0.521 (0.437-0.607) | 0.59 | 3.53E-05 | 0.704 (0.623-0.778) | 1.42 | 1.08E-11 | 0.804 (0.722-0.87) | 1.36 | 9.68E-13 | 0.916 (0.866-0.952) |
| CTRL | preAD | 0.09 | 1 | 0.5 (0.372-0.629) | 0.49 | 0.14 | 0.748 (0.648,0.833) | 0.97 | 0.0014 | 0.741 (0.627-0.837) | 0.93 | 0.0039 | 0.728 (0.604-0.832) |
| CTRL | MCI-AD | 0.09 | 1 | 0.527 (0.43-0.622) | 0.58 | 5.85E-04 | 0.534 (0.433,0.633) | 1.36 | 9.97E-09 | 0.811 (0.73-0.881) | 1.31 | 1.47E-09 | 0.929 (0.876-0.965) |
| CTRL | AD-dem | 0.17 | 1 | 0.521 (0.412-0.627) | 0.62 | 1.24E-04 | 0.573 (0.474,0.664) | 1.39 | 3.24E-08 | 0.82 (0.726-0.895) | 2.50 | 2.34E-08 | 0.965 (0.914-0.992) |

**Table S4. Effect size (Cohen’s d), p-values adjusted for age, gender and FDR and age-adjusted AUC for pairwise comparisons between AD clinical stages and CTRL for each of the plasma biomarker measured with Lumipulse® G.**


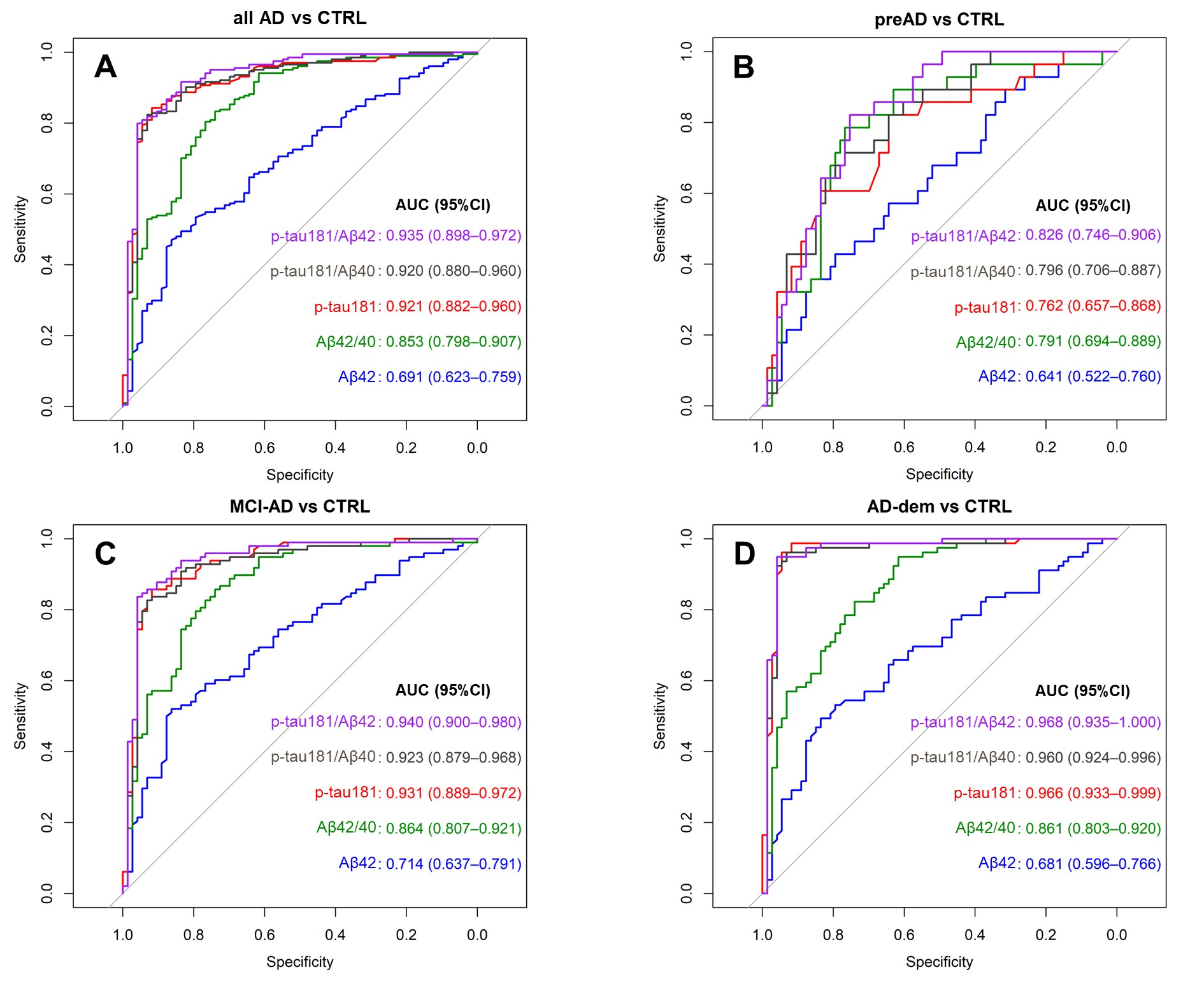
**Figure S7. ROC curves quantifying the ability of plasma Aβ42, Aβ42/40, p-tau181, p-tau181/Aβ40, and p-tau/Aβ42 in differentiating AD stages from CTRL**. Areas under the ROC curves (AUC) are displayed for each comparison, i.e., *all* AD vs CTRL (A), preAD vs CTRL (B), MCI-AD vs CTRL (C), and AD-dem vs CTRL (D), together with their 95% confidence interval calculated by generating 2000 bootstrap replicates.


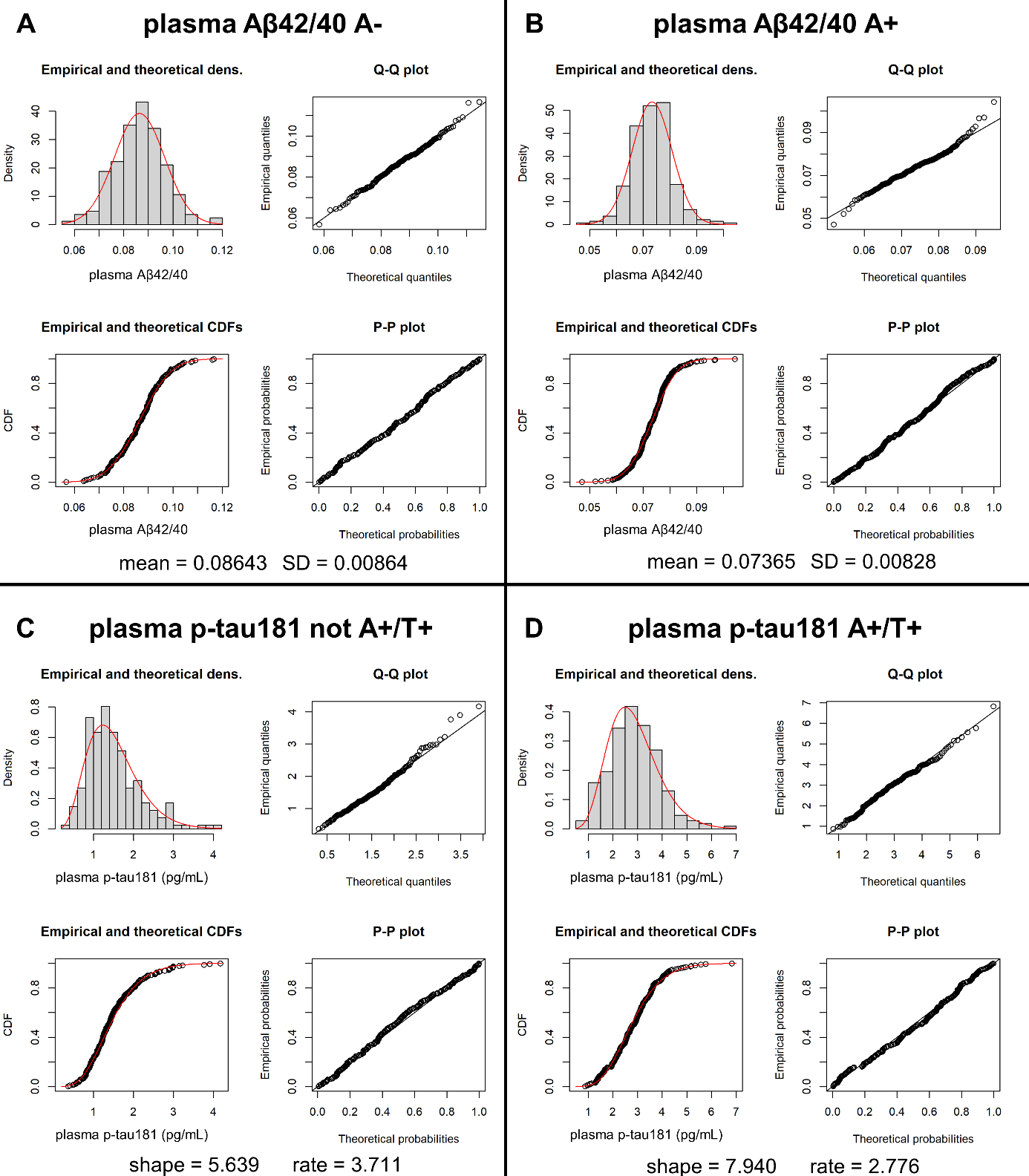


**Figure S8. Fitting details of the univariate models.** Histograms, quartile-quartile (Q-Q) plots, empirical and estimated cumulative density functions (CDF) and probability-probability (P-P) plots are reported for each fitted probability density function. A-B) Plasma Aβ42/40 values of subjects with A- and A+ CSF profiles were fitted by means of normal distributions. C-D) Plasma p-tau181 values of subjects without A+/T+ and with A+/T+ CSF profiles were fitted by means of gamma distributions.


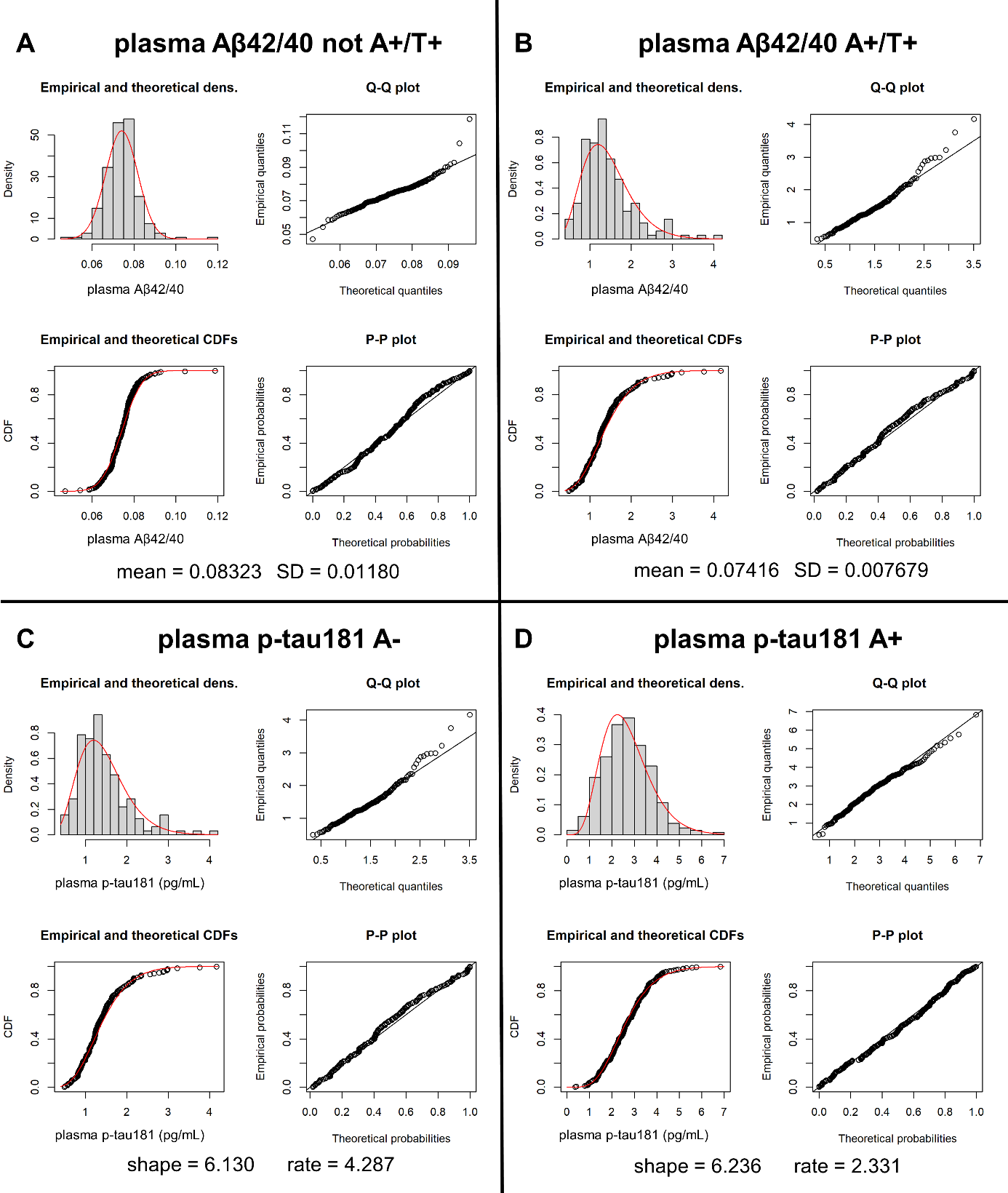


**Figure S9 Fitting details of complementary univariate models used to compute bivariate models**. Histograms, quartile-quartile (Q-Q) plots, empirical and estimated cumulative density functions (CDF) and probability-probability (P-P) plots are reported for each fitted probability density function. A-B) Plasma Aβ42/40 values of subjects without A+/T+ and with A+/T+ CSF profiles were fitted by means of normal distributions. C-D) Plasma p-tau181 values of subjects with A- and A+ CSF profiles were fitted by means of gamma distributions.

|  | **A** | CSF profile | | |  |
| --- | --- | --- | --- | --- | --- |
|  |  | **A-/T±** | **A+/T-** | **A+/T+** |  |
| Plasma profile | **A-/T±** | 115 | 4 | 14 |  |
|  | **A+/T-** | 23 | 22 | 22 |  |
|  | **A+/T+** | 21 | 20 | 179 |  |
|  | N | 159 | 46 | 215 |  |

|  | **B** | CSF profile | | |  |
| --- | --- | --- | --- | --- | --- |
|  |  | **A-/T±** | **A+/T-** | **A+/T+** |  |
| Plasma profile | **A-/T±** | 84 | 3 | 5 |  |
|  | **A+/T-** | 0 | 1 | 0 |  |
|  | **A+/T+** | 4 | 8 | 109 |  |
|  | N | 88 | 12 | 114 |  |

**Table S5. Confusion matrices summarizing classification results for trinomial prediction of CSF profile from plasma Aβ42/40 and p-tau181.** The serial application of the two binomial bivariate models allowed for the prediction of the A-/T±, A+/T-, and A+/T+ CSF profiles in all the KD- samples from the UNIPG cohort (A) and for the subset of samples whose prediction probabilities from bivariate models where lower than 0.3 or higher than 0.7 (B).

**R script to apply univariate and bivariate binomial and trinomial classification models**

# function to apply the bivariate model to calculate the A+ probability

A_plus_bivariate <- function(abratio,ptau) {

mean_Ap_abratio<-0.073651

sd_Ap_abratio<-0.008281

mean_An_abratio<-0.086432

sd_An_abratio<-0.010181

shape_An<-6.1299

shape_Ap<-6.2357

rate_Ap<-2.331

rate_An<-4.2872

dens1A <- dgamma(ptau, shape = shape_Ap, rate = rate_Ap)

dens2A <- dgamma(ptau, shape = shape_An, rate = rate_An)

probAp_ptau <- dens1A / (dens1A + dens2A)

dens1B<-dnorm(abratio, mean_Ap_abratio,sd_Ap_abratio)

dens2B<-dnorm(abratio, mean_An_abratio,sd_An_abratio)

probAp_abratio <- dens1B / (dens1B + dens2B)

k=0.663

prob_Ap=k*probAp_abratio+(1-k)*probAp_ptau

return(prob_Ap)

}

# function to apply the bivariate model to calculate the A+/T+ probability

A_plus_T_plus_bivariate <- function(abratio,ptau) {

shape_ApTp<-7.9404

rate_ApTp<-2.7764

shape_nApTp<-5.6388

rate_nApTp<-3.7114

mean_ApTp<-0.0741615

sd_ApTp<-0.0076786

mean_nApTp<-0.083231

sd_nApTp<-0.011796

dens1A <- dgamma(ptau, shape = shape_ApTp, rate = rate_ApTp)

dens2A <- dgamma(ptau, shape = shape_nApTp, rate = rate_nApTp)

probApTp_ptau <- dens1A / (dens1A + dens2A)

dens1B<-dnorm(abratio, mean_ApTp,sd_ApTp)

dens2B<-dnorm(abratio, mean_nApTp,sd_nApTp)

probApTp_abratio <- dens1B / (dens1B + dens2B)

k=0.1672241

prob_ApTp=k*probApTp_abratio+(1-k)*probApTp_ptau

return(prob_ApTp)

}

# functions to apply trinomial classification from binomial bivariate models

AT_plasma <- function(Ap_biv,ApTp_biv) {

AT_profile<-ifelse(ApTp_biv>0.5,"A+/T+",ifelse(Ap_biv>0.5,"A+/T-","A-/Tx"))

return(AT_profile)

}

ApTp_prob <- function(Ap_biv,ApTp_biv) {

prob_classApTp<-ApTp_biv*1/(ApTp_biv+(1-ApTp_biv)*Ap_biv+(1-ApTp_biv)*(1-Ap_biv))

return(prob_classApTp)

}

ApTm_prob <- function(Ap_biv,ApTp_biv) {

prob_classApTm<-(1-ApTp_biv)*Ap_biv*1/(ApTp_biv+(1-ApTp_biv)*Ap_biv+(1-ApTp_biv)*(1-Ap_biv))

return(prob_classApTm)

}

AmTx_prob <- function(Ap_biv,ApTp_biv) {

prob_classAmTx<-(1-ApTp_biv)*(1-Ap_biv)*1/(ApTp_biv+(1-ApTp_biv)*Ap_biv+(1-ApTp_biv)*(1-Ap_biv)

return(prob_classAmTx)

}

# function to apply the univariate model based on plasma Aβ42/40 to calculate the A+ probability

A_plus_univariate <- function(abratio) {

mean_Ap_abratio<-0.073651

sd_Ap_abratio<-0.008281

mean_An_abratio<-0.086432

sd_An_abratio<- 0.010181

dens1B<-dnorm(abratio, mean_Ap_abratio,sd_Ap_abratio)

dens2B<-dnorm(abratio, mean_An_abratio,sd_An_abratio)

probAp_abratio <- dens1B / (dens1B + dens2B)

return(probAp_abratio)

}

# function to apply the univariate model based on plasma p-tau181 to calculate the A+/T+ probability

A_plus_T_plus_univariate <- function(ptau) {

shape_ApTp<-7.9404

rate_ApTp<-2.7764

shape_nApTp<-5.6388

rate_nApTp<-3.7114

dens1A <- dgamma(ptau, shape = shape_ApTp, rate = rate_ApTp)

dens2A <- dgamma(ptau, shape = shape_nApTp, rate = rate_nApTp)

probApTp_ptau <- dens1A / (dens1A + dens2A)

return(probApTp_ptau)

}

#code to apply functions. We suggest to apply bivariate models when both plasma Aβ42/40 and p-tau181 measured with Lumipulse® are available, univariate models when just one of these biomarkers are available

library(readxl)

data <- read_excel("add your here your dataset")

abratio<-data$abratio #add here plasma Aβ42/40 ratio from your data, you can also add a single numeric value

ptau<- data$ptau #add here plasma p-tau181 from your data, you can also add a single single numeric value

# abratio<-0.075 #example

# ptau<-2.5 #example

#application of bivariate models

Ap_biv<-A_plus_bivariate(abratio,ptau)

ApTp_biv<-A_plus_T_plus_bivariate(abratio,ptau)

#application of trinomial classification from bivariate models

AT_profile_plasma<-AT_plasma(Ap_biv,ApTp_biv)

out<-data.frame(Ap_biv)

out$ApTp_biv<-ApTp_biv

out$plasma_profile<-AT_profile_plasma

out$prob_ApTp<-ApTp_prob(Ap_biv,ApTp_biv)

out$prob_ApTm<-ApTm_prob(Ap_biv,ApTp_biv)

out$prob_AmTx<-AmTx_prob(Ap_biv,ApTp_biv)

write.csv(out, "out_bivariate.csv", row.names=FALSE) #saves the results of bivariate models in a CSV file

# application of univariate models

Ap_uni<-A_plus_univariate(abratio)

ApTp_uni<-A_plus_T_plus_univariate(ptau)

out<-data.frame(Ap_uni)

out$ApTp_uni<-ApTp_uni

write.csv(out, "out_univariate.csv", row.names=FALSE) #saves the results of univariate models in a CSV file

**References**

[1] Jack CR, Bennett DA, Blennow K, Carrillo MC, Dunn B, Haeberlein SB, et al. NIA-AA Research Framework: Toward a biological definition of Alzheimer’s disease. Alzheimers Dement 2018;14:535–62. https://doi.org/10.1016/j.jalz.2018.02.018.

[2] Postuma RB, Berg D, Stern M, Poewe W, Olanow CW, Oertel W, et al. MDS clinical diagnostic criteria for Parkinson’s disease. Mov Disord 2015;30:1591–601. https://doi.org/10.1002/mds.26424.

[3] McKeith IG, Boeve BF, Dickson DW, Halliday G, Taylor J-P, Weintraub D, et al. Diagnosis and management of dementia with Lewy bodies: Fourth consensus report of the DLB Consortium. Neurology 2017;89:88–100. https://doi.org/10.1212/WNL.0000000000004058.

[4] Emre M, Aarsland D, Brown R, Burn DJ, Duyckaerts C, Mizuno Y, et al. Clinical diagnostic criteria for dementia associated with Parkinson’s disease. Mov Disord 2007;22:1689–707; quiz 1837. https://doi.org/10.1002/mds.21507.

[5] Litvan I, Goldman JG, Tröster AI, Schmand BA, Weintraub D, Petersen RC, et al. Diagnostic criteria for mild cognitive impairment in Parkinson’s disease: Movement Disorder Society Task Force guidelines. Mov Disord 2012;27:349–56. https://doi.org/10.1002/mds.24893.

[6] Gorno-Tempini ML, Hillis AE, Weintraub S, Kertesz A, Mendez M, Cappa SF, et al. Classification of primary progressive aphasia and its variants. Neurology 2011;76:1006–14. https://doi.org/10.1212/WNL.0b013e31821103e6.

[7] Rascovsky K, Hodges JR, Knopman D, Mendez MF, Kramer JH, Neuhaus J, et al. Sensitivity of revised diagnostic criteria for the behavioural variant of frontotemporal dementia. Brain 2011;134:2456–77. https://doi.org/10.1093/brain/awr179.

[8] Delaby C, Teunissen CE, Blennow K, Alcolea D, Arisi I, Amar EB, et al. Clinical reporting following the quantification of cerebrospinal fluid biomarkers in Alzheimer’s disease: An international overview. Alzheimer’s & Dementia 2022;18:1868–79. https://doi.org/10.1002/alz.12545.

[9] Zwan MD, van der Flier WM, Cleutjens S, Schouten TC, Vermunt L, Jutten RJ, et al. Dutch Brain Research Registry for study participant recruitment: Design and first results. Alzheimers Dement (N Y) 2021;7:e12132. https://doi.org/10.1002/trc2.12132.

[10] van der Flier WM, Pijnenburg YAL, Prins N, Lemstra AW, Bouwman FH, Teunissen CE, et al. Optimizing patient care and research: the Amsterdam Dementia Cohort. J Alzheimers Dis 2014;41:313–27. https://doi.org/10.3233/JAD-132306.

[11] van der Flier WM, Scheltens P. Amsterdam Dementia Cohort: Performing Research to Optimize Care. J Alzheimers Dis 2018;62:1091–111. https://doi.org/10.3233/JAD-170850.
